# Spatial ecostructural modelling of endometrial cancer identifies the key role of CD90 + CD105 + endothelial cells in tumour heterogeneity and predicts disease recurrence

**DOI:** 10.1186/s40164-025-00724-6

**Published:** 2025-11-17

**Authors:** Di Wu, Cinian Lv, Zhifeng Yan, Luyang Zhao, Lian Li, Mingxia Ye, Mingyang Wang, Qingzhi Zhai, Nan Wang, Zheng Wang, Yuanguang Meng, Mingxia Li

**Affiliations:** 1 Senior Department of Obstetrics and Gynecology, Chinese PLA General Hospital, No. 5, Nanmencang, Dongsishitiao, Dongcheng District, Beijing, 100700 China; 2 School of Medicine, Nankai University, No. 94, Weijin Road, Nankai District, Tianjin, 300071 China; 3 School of Biomedical Engineering, Tsinghua University, No.30 Shuangqing Road, Haidian District, Beijing, 100084 China

**Keywords:** Endometrial cancer, Molecular subtypes, Tumour microenvironment, Single-cell spatial analysis

## Abstract

**Background:**

Current therapeutic strategies for endometrial cancer are mainly based on aggressive histological types and molecular subtypes. However, ignoring the spatial distribution of immune/stromal cells fails to account for the heterogeneity of the local tumour microenvironment, leading to biased prediction of treatment response. The goal of precision medicine is to delineate the biological characteristics of local functional units based on molecular labelling, which adequately reflects spatially adaptive changes during treatment or metastasis.

**Methods:**

Single-cell resolution analysis of 40 endometrial cancer cases across four molecular subtypes was performed using imaging mass cytometry (IMC) to quantify the frequency, spatial distribution, and intercellular crosstalk of distinct immune and stromal cell populations. These ecosystem-level features were systematically correlated with clinical features and outcomes, including treatment response and survival. We further identified CD90 + clusters as key regulators of macrophage polarization and T-cell infiltration dynamics, with flow cytometry used to validate their functional role in tumour subtype specification and microenvironmental remodelling. Finally, machine learning-based spatial phenotyping was employed to construct molecular subtype-specific signatures and a highly accurate recurrence prediction model for high-risk endometrial cancer.

**Results:**

Single-cell profiling revealed that CD90 + clusters constitute a critical immunomodulatory component within the tumour microenvironment, demonstrating significant enrichment in macrophage differentiation pathways and serving as key mediators of intercellular signalling networks. Furthermore, computational models integrating functional molecular signatures with cell–cell interaction profiles demonstrated high predictive accuracy for both molecular subtyping and recurrence risk stratification in patients with endometrial carcinoma.

**Conclusions:**

Our study establishes a spatial eco-context framework for molecular subtypes of endometrial cancer by integrating single-cell spatial multiomics data. This approach enables high-resolution mapping of tumour-immune-stromal interaction networks and reveals novel targets for personalized therapeutic strategies.

**Supplementary Information:**

The online version contains supplementary material available at 10.1186/s40164-025-00724-6.

## Background

In 2020, endometrial cancer was the second most common malignancy in the female reproductive tract globally, accounting for 4.3% of all cases [1]. Moreover, an estimated 97,370 endometrial cancer deaths occur globally, with an age-standardized mortality rate (ASMR) of 1.8 (1/100,000), resulting in a significant health burden [1]. Endometrioid endometrial carcinoma (EEC) is the most common histological subtype of endometrial cancer, accounting for approximately 70–80% of cases [2]. EEC is a highly heterogeneous group of tumours with unique cellular and molecular characteristics. International societies have recommended updated risk stratification strategies to guide adjuvant therapy, including selected single prognostic markers and molecular-based classification methods [3, 4]. With the gradual development and clinical application of molecular typing, accurate stratification, prognosis assessment and treatment guidance are needed. The consensus of a large number of studies is that POLE hypermutated endometrial cancer patients have the best prognosis, especially for some high-grade (G3) patients. If no other intermediate and high-risk factors are present, they can be treated without adjuvant therapy to avoid overmedication. Patients with MSI-H-type cancers have a better response to immune-targeted therapy, a relatively good prognosis, and can be scientifically stratified and accurately treated. Patients with CNH-type cancer have the poorest prognosis and need to undergo comprehensive treatment to avoid insufficient treatment, which affects their survival. At present, the clinical value of the CNL type has not been clarified, and in-depth research is needed to re-stratify it. While molecular subtyping provides a more nuanced and biologically informed approach to risk stratification than traditional clinicopathological factors, it has certain limitations. For example, molecular subtypes are not completely mutually exclusive, and some overlap or intermediate phenotypes may complicate the risk stratification process. Molecular subtyping may not fully capture extensive intra-tumoural heterogeneity, leading to incomplete representation of the tumour ecosystem. Among the molecular subtypes of endometrial cancer, the copy-number low (CNL) subtype presents distinct challenges in terms treatment and prognosis [5]; its genomic stability and specific hormone-dependent characteristics differentiate it from more aggressive subtypes, highlighting the need for dedicated research to elucidate the complexities of its biology and the implications for tumour behaviour. Additionally, molecular subtypes alone may not always accurately predict clinical outcomes or treatment response, as they do not account for the influential roles of the surrounding stromal, immune, and vascular components. Furthermore, contextual and spatial information cannot be provided by molecular subtypes alone, affecting our understanding of disease mechanisms and, in particular, clinical expectations for immunotherapy [6]. Therefore, recommendations for the treatment of early-stage endometrial cancer remain controversial [7–9]. Many negative and excessive medical practices cannot be standardized [10, 11].

The tumour microenvironment (TME) is a major source of EEC heterogeneity and influences disease progression and response to therapy [12]. The TME encompasses noncancer cells, the extracellular matrix, and signalling molecules that surround and interact with tumour cells [13, 14]. By integrating molecular subtyping and TME information, we can gain more multidimensional insights into disease progression mechanisms and reveal new therapeutic vulnerabilities. However, traditional bulk tissue analysis has many drawbacks [15]. Traditional bulk tissue analysis provides an average signal from a heterogeneous sample, potentially masking important biological differences and cellular dynamics and revealing the spatial distribution of different cell types and their interactions within the tumour microenvironment [16]. Moreover, with the loss of functional states of cells, bulk tissue analysis might lead to erroneous conclusions about the presence or absence of specific proteins or pathways that are critical for understanding tumour behaviour.

Hence, we employed imaging mass spectrometry (IMC) to obtain high-resolution spatial proteomics profiles of the four subtypes of early-stage ECC. TME studies in early-stage endometrial cancer are an ideal model for exploring the mechanisms of tumour-microenvironment interactions due to its low heterogeneity, well-defined driver mutations, active immune infiltration, and clinical tractability and are particularly helpful for revealing the early events of immune escape and targeted therapies, which can provide the basis for precision therapeutic strategies. The revolutionary technology used in the study of the tumour microenvironment is a combination of mass spectrometry and imaging that enables the spatial distribution of multiple proteins and other biomolecules to be analysed at the single-cell level in their native environments, integrating them to generate quantitative data, making it an invaluable tool for advancing our understanding of tumour biology and the immune landscape [17].

The main focus of this study was to elucidate the spatial proteomic features of endometrial cancer (EEC) samples with different molecular types using advanced imaging mass spectrometry (IMC) techniques. In addition, we seek to link these tumour microenvironment (TME) features to patient prognosis to better analyse the personal need for adjuvant therapies after staging surgery. Ultimately, we intend to develop a predictive tool that combines spatial proteomic data with machine learning techniques to predict tumour recurrence, thereby not only deepening our understanding of the mechanisms of tumour recurrence but also enhancing personalised treatment options for EEC patients.

## Materials and methods

### Clinical cohort and ethics

A total of 40 patients with EEC were included in this study, with a follow-up period from May 2021 to August 2024. The inclusion criteria were patients who were diagnosed with endometrioid adenocarcinoma, which is the gold standard of surgical pathology, without any preoperative treatment and had imaging assessment to define stage I (IA or IB). The exclusion criterion was a combination of other malignancies or psychiatric disorders that prevented cooperation. All samples obtained were paraffin samples obtained from the initial standardised staging procedure. Clinical information for all patients is shown in the Supplementary Table. Tissue microarrays were constructed from a 1 mm^2^ core of tissue selected from surgical tumour specimens, with 2 tissue chips per patient.

This study adhered to the ethical guidelines set forth by the Ethics Committee of the General Hospital of the People’s Liberation Army. The ethical review was passed under the number S2024-422-01. Written informed consent was obtained from all participants.

### IMC section preparation

Formalin-fixed, paraffin-embedded (FFPE) tissue samples from patients with endometrial cancer (EEC) were sectioned at 4 µm using the HistoCore MULTICUT system (Leica, Germany). These sections were heated at 70 °C for 1 h, followed by complete deparaffinisation with xylene. Sections were immersed in decreasing concentrations of ethanol solutions (95, 85 and 75%) at ambient temperature and then placed in sodium citrate buffer and heated at 100 °C for 30 min for antigen recovery. After cooling to room temperature, the sections were washed twice in PBS-TB (0.5% Tween-20 and 1% BSA) for 5 min each time. The sections were blocked using SuperBlock (37,515; Thermo Fisher Scientific) for 30 min at room temperature. The sections were then washed three times with PBS-TB and incubated overnight at 4 °C with a cocktail of antibodies containing metal-labelled antibodies. Antibodies were conjugated to metals using the Maxpar® X8 Antibody Labelling Kit (Fluidigm). After incubation, the sections were washed three times with PBS-TB. The nuclei were labelled with Intercalator-Ir (201192B, Fluidigm) solution in PBS-TB (1.25 µM) for 30 min at room temperature, followed by two washes with PBS-TB and a final rinse with ddH2O. Each group consisted of 10 samples with two regions of interest (ROIs) per sample.

### Cell segmentation

The images were first denoised and enhanced for contrast before cell segmentation was performed using the pre-trained TissueNet algorithm. This algorithm delineates cell nuclei typically stained with DAPI and identifies cell membranes through additional channels to define individual cell boundaries.

### IMC data transformation and annotation

After integrating all the samples and correcting different batches using the Harmony algorithm, a more homogeneous subpopulation distribution can be observed. Cell clustering was subsequently performed using Rphenograph (version 0.99.1). More refined clustering was conducted using more markers to delineate lymphocytes (including FoxP3, CD3, CD4, CD8, CD127, CD45, CD45RO, CD38, CD16, CD20, CD57, and granzyme B), epithelial cells (including E-cadherin and pancytokeratin), myeloid cells (including S100A9, CD11c, and CD163), endothelial cells (CD31), stromal cells, and various cytokines (HIF1a, Ki67, CXCR4, SDF1, TNFα, and IL-1b) and immune checkpoints (PD-L1 and PD-1). Each subgroup was isolated from the total population and reclustered using the same protocol. The resulting clusters were visualised in a heatmap to assist in cell type annotation.

### Region and milieu identification

We employed the patchDetection function from the imcRtools package to assess local cell clustering. Three primary regions were defined as follows: the epithelial region with positive expression of pancytokeratin, the fibrous region with positive expression of collagen 1, and the immune region with positive expression of CD45. A minimum of 20 cells was required to define a region, with a maximum distance of 20 µm between relevant cells. Adjacent regions were merged for downstream analysis. The regions were then expanded by 20 µm, resulting in small areas equivalent to approximately two cell diameters.

### NMF analysis

#### Proteomics data acquisition

Proteomics techniques were used to generate a high-dimensional matrix, where rows represent proteins and columns represent samples.

#### Data preprocessing

The proteomics data were normalised to reduce technical variability and ensure comparability across samples. Low-abundance proteins or noise were removed to focus on biologically relevant features.

#### Matrix formation

A non-negative matrix V was constructed where Vij represents the abundance of protein i in sample j.

#### NMF decomposition (factorization)

The matrix V was decomposed into two non-negative matrices, W (basis matrix) and H (coefficient matrix), using the following relationship: V ≈ WH (W represents the latent factor protein signatures or profiles; H indicates the contribution of each factor to the original samples.)

#### Determining the number of factors

Methods were used to select the optimal number of components for analysis.

#### Interpretation of results

The basis matrix W was analysed to identify distinct protein signatures associated with different cellular populations or tumour microenvironment characteristics. Clustering techniques and visualisation tools (e.g., heatmaps) were used to interpret and present the results, highlighting spatial and functional relationships between protein signatures.

### Cell–cell pairwise interaction and cell neighbourhood (CN) identification

A permutation test analysis of spatial single-cell was conducted to identify significant pairwise interaction and avoidance behaviours. Cells located within a 6-pixel radius (6 µm) were deemed to be interacting. A p value of less than 0.01 was considered to indicate a significant relationship. A "window" capture strategy was employed to analyse cell neighbourhoods. For each cell, a defined radius (e.g., 20 µm) was established to identify neighbouring cells, which resulted in the creation of frequency vectors representing the types and counts of neighbouring cells. Initial cell clustering was performed using the MiniBatchKMeans clustering algorithm implemented in the Scikit-learn library (version 0.24.2), with a default batch size of 100 and random seed set to 0. Subsequent analysis utilized MiniBatchKMeans (version 1.1.2) with a batch size of 1,024. Each cell was assigned to a neighbourhood based on its defining window. The prevalence of each neighbourhood within the core was normalised such that the total prevalence equalled 100%. Z scores were calculated for these values to facilitate comparisons between neighbourhoods, specifically focusing on those with z scores above or equal to 0 and below 0. The results of the neighbourhood analysis were visualized using heatmaps to illustrate the distribution and interactions of cell types within the tissue.

### Multivariate modelling and variable importance

We extracted three different sets of features for each ROI, including cell population frequency, cell–cell interaction, and functional marker expression. Seventy percent of the data were randomly selected as the training set, and 30% of the data were used as the validation set. The number of variables randomly sampled as candidates at each split was selected based on the model misclassification rate predicted by the out-of-bag samples, and a random forest classifier was fitted to the training set. Next, the validation set data were used to further evaluate the classifier performance. This process was repeated 1000 times to generate specific features, and the importance of the features to the model was evaluated by mean decrease accuracy, where a larger value indicates greater importance of the variable. Finally, the results of feature importance can be visualised or ranked by importance to better understand which features play a key role in the prediction model. We used the AUC statistic to evaluate the predictive efficiency of the identified features.

### Flow cytometry technique

Single-cell suspensions were prepared from tumour samples and stained with fluorochrome-conjugated antibodies against CD90, CD105, and CD31. CD31 expression was used to confirm the identity of the endothelial cells. Following staining, the samples were washed to remove any unbound antibodies. Stained cells were then analysed using flow cytometry to accurately quantify the CD90 + CD105 + double-positive endothelial cell population.

### Migration assay

For the Transwell migration assay, THP-1 cells were seeded in the top chamber of a Transwell insert with a porous membrane, whereas the bottom chamber included CD90 + CD105 + endothelial cells. After incubation, the cells on the top or bottom of the membrane were fixed, stained, and quantified using image analysis.

### Cell proliferation

CCK-8 and colony formation assays were utilised to assess cell viability and proliferation. For the CCK-8 assay, cells were seeded in plates and incubated under the experimental conditions. After the desired timepoint, the CCK-8 reagent was added, and the absorbance at 450 nm was measured using a spectrophotometer, which correlated with the number of viable cells.

### Western blotting

Proteins were extracted from cells with the addition of protease inhibitors to prevent degradation during the lysis process. Protein concentrations were then quantified using the BCA method to ensure accurate results. Target proteins were separated via SDS-PAGE, followed by incubation with primary and secondary antibodies. The specific protein antibodies used in this study included anti-iNOS (ab283655), anti-IRF5 (ab181553), Arginase1 (PA5-18392), CD206 (JF0953), and β-actin (ab8226).

### ELISA assay

The cell lysates were first collected and centrifuged at 4 °C to remove impurities. Specific precoated ELISA kits were selected according to the target cytokines (M1 type-associated TNF-α and M2 type-associated IL-10). For the experiment, the standard curve was constructed by gradient dilution of the standards, and the samples were added to 96-well plates after appropriate dilution and incubated for 2 h at room temperature. After the samples were washed, biotinylated detection antibody was added and incubated for 1 h. After the samples were washed three times with PBS-T, HRP-labelled streptavidin was added and allowed to react for 30 min. TMB substrate was used to develop the colour for 15 min, after which the reaction was terminated, and the absorbance was measured immediately by an enzyme marker at 450 nm. The concentration of the samples was calculated from the standard curve, and duplicate wells and blank controls were used to ensure the reliability of the data. Note that all the steps were performed under the condition of avoiding light.

### Statistical analysis

All image downstream analyses were conducted using R software. Statistical analyses were performed using R Studio (version 4.2.2). Data are presented as the mean ± standard error of the mean (s.e.m.) or mean ± standard deviation (s.d.), with *P* < 0.05 considered significant unless stated otherwise. The statistical tests employed are detailed in the figure legends, and survival data were analysed using the survival package.

## Results

### Inclusion of patients in the retrospective study

**Fig. 1:**
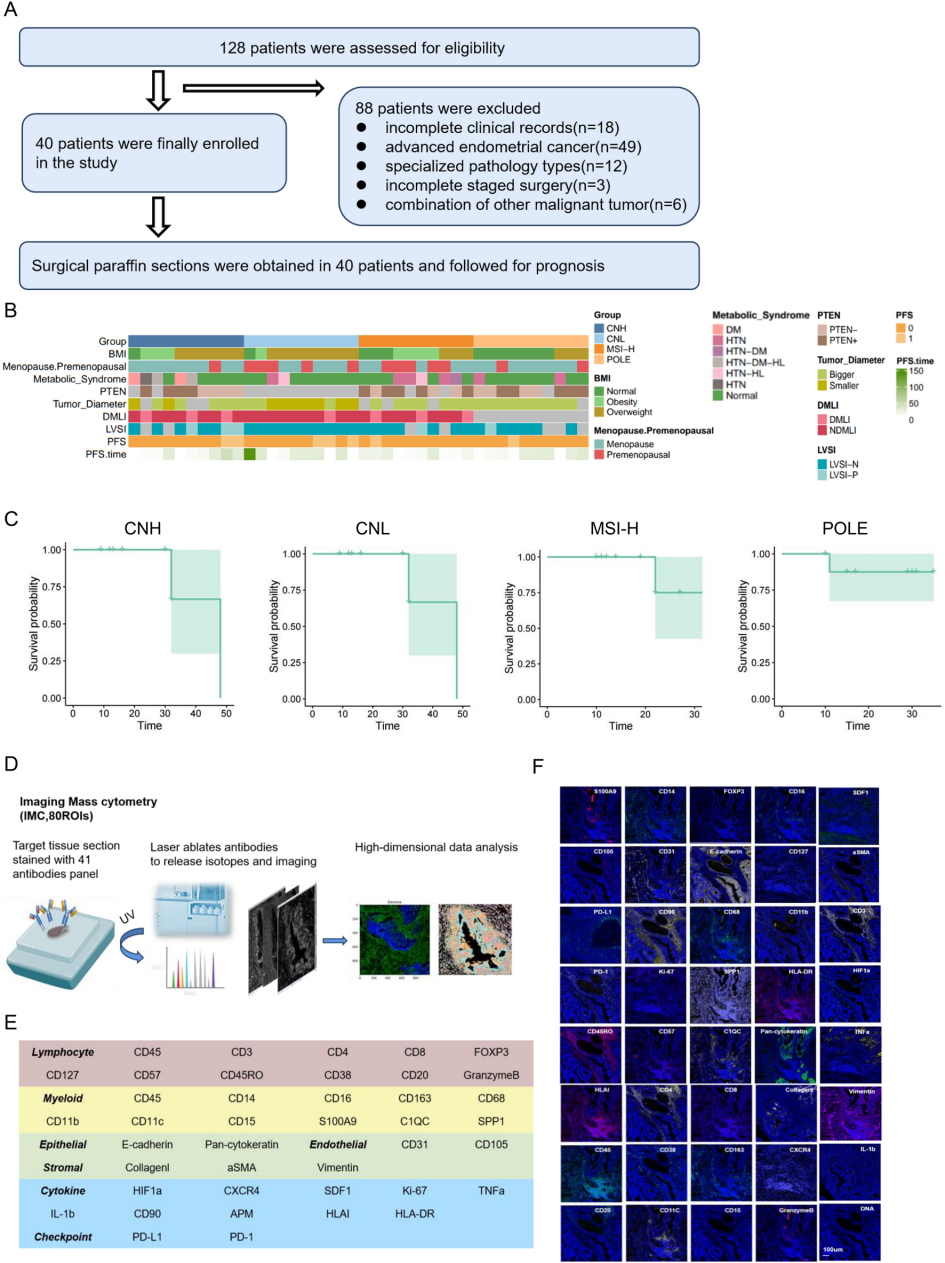
Patient inclusion and collection of specimens.** A** Patient inclusion process for this retrospective study. **B** Clinical characteristics and tumour pathology of the 40 included patients. BMI (body mass index) in the figure is the number obtained by dividing the weight in kilograms by the square of the height in metres, which is a standard commonly used internationally to measure the degree of fatness and thinness of the human body; PTEN is a common gene mutation in endometrioid carcinoma; and DMLI (deep myometrial infiltration) and LVSI (lymphovascular interstitial infiltration) are both high-risk pathological features of the tumour. PFS (progression-free survival) is an indicator for evaluating prognosis. **C** Survival curves were plotted according to the molecular subtypes of the included patients, which were CNH, CNL, MSI-H and POLE. The specific survival indicator was progression-free survival in months. **D** Schematic diagram of the IMC process applied in this study. **E** Panel of antibodies involved in IMC detection technology, showing both lineage and major functional markers for cell type identification. **F** This panel presents a representative single-colour plot from imaging mass cytometry (IMC), illustrating the expression of a specific marker across tissue sections. This visualisation aids in identifying the spatial distribution and intensity of the marker within the cellular microenvironment

The primary aim of this study was to gain insight into the tumour microenvironment of EEC patients with four TCGA molecular subtypes and, in particular, to identify differences in the spatial characteristics of recurrent patients. A total of 128 patients were assessed for eligibility for this study from May 2021 to August 2023. Forty patients met the inclusion criteria (Fig. 1A). All 40 patients underwent standard staging surgery at the gynaecology department of the First Medical Centre of the Chinese People's Liberation Army General Hospital, and all of them achieved complete resection of the tumour (R0 resection), demonstrating no residual lesions under the microscope. No statistically significant differences were observed in age, body mass index, menopausal status at diagnosis, metabolic comorbidities, tumour stage and differentiation among the patients carefully assessed for the four subtypes of endometrial cancer, as detailed in Fig. 1B. Based on basic demographic and clinical characteristics of the patients, the median age was 56 years, ranging from 38 to 85 years. The patients were diagnosed with early stage endometrial cancer with or without deep myometrial infiltration, which manifested mainly as irregular vaginal bleeding or postmenopausal vaginal bleeding. Additionally, the survival curves illustrate the progression-free survival (PFS) for the cohort of 40 patients (Fig. 1C). Preserved paraffin-embedded tumour specimens were collected, and 41 specific proteins were comprehensively using IMC technology to characterize the spatial distribution and interaction patterns of the cells of the endometrial tumour microenvironment while preserving ecological structural integrity (Fig. 1D). We optimised a 41-plex IMC panel that encompassed 39 protein markers along with DNA (Fig. 1E). Our IMC panel encompasses markers for epithelial cells, endothelial cells, stromal cells, and innate and adaptive immune lineages, such as the chemokine receptor CXCR4 and its ligand CXCL12 (SDF-1); the proliferation marker Ki-67; hypoxia-inducible Factor 1 subunit alpha (HIF1a); and immune checkpoint-related markers, including PD-1 and programmed death ligand 1 (PD-L1). Each antibody was validated by immunohistochemistry (IHC) before binding to heavy metals, and each metal-conjugated antibody was further validated using IMC (Fig. 1F).

### Comprehensive EEC tumour immune microenvironment at single-cell resolution

**Fig. 2:**
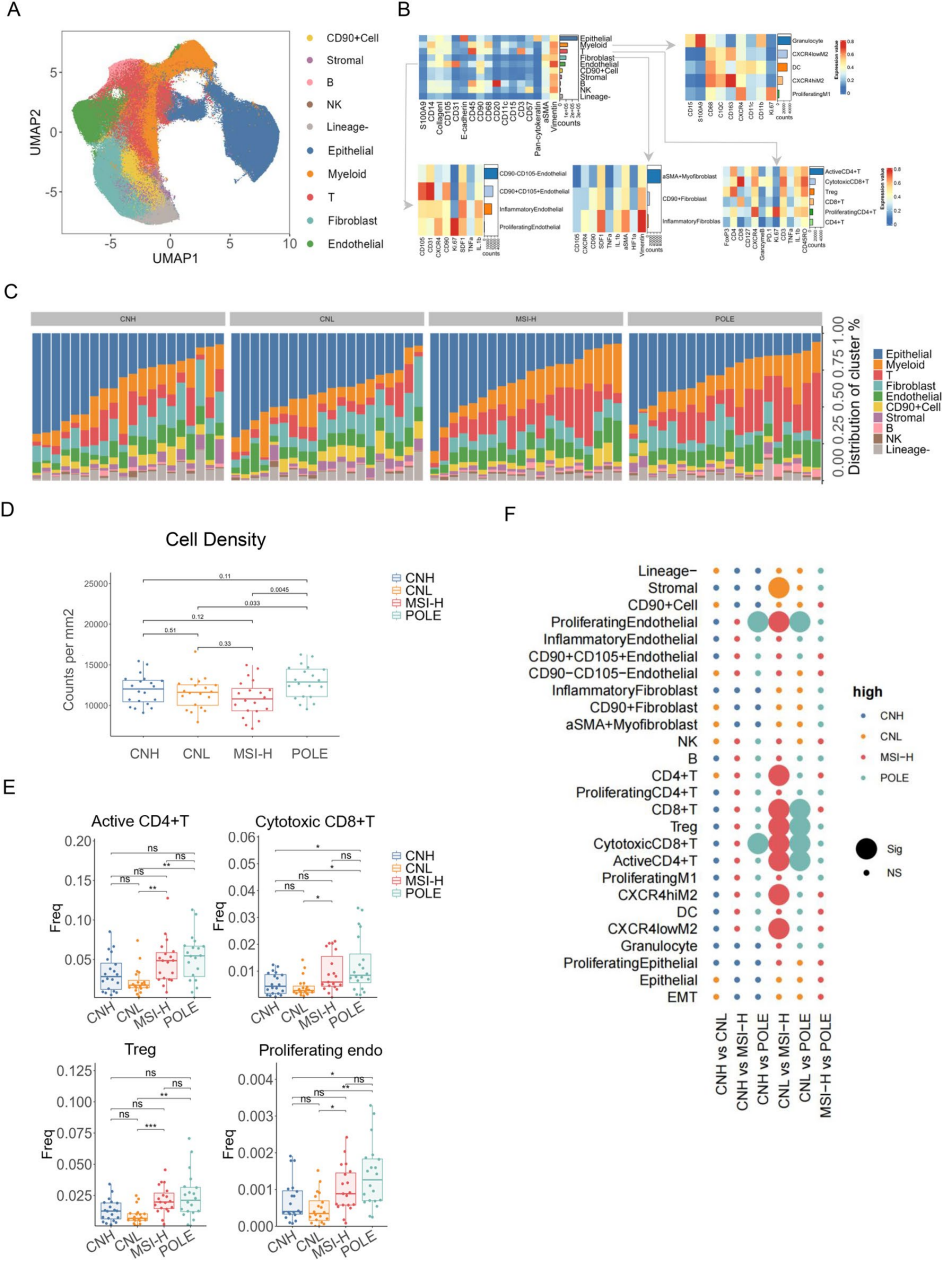
Comprehensive immune microenvironment of EEC tumours at single-cell resolution.** A.** Uniform Manifold Approximation and Projection. UMAP visualisation reduces multidimensional data to a two-dimensional space, facilitating the identification of clusters and patterns within the complex cellular landscape of the tumour microenvironment (TME). **B** This dual-layer heatmap illustrates major and detailed cellular phenotypes within the tumour microenvironment (TME). The upper-left section displays the primary cell types identified, while the other sections provide a more granular view of subtypes, highlighting their expression profiles across various samples or conditions. The colour of each tile indicates the mean expression level of the corresponding marker in each cell type. **C** Waterfall plot depicting the distribution of stromal and immune cell types across molecular subgroups. **D** Statistical analysis of overall cell density between groups of different molecular subtypes (CNH = copy number high, CNL = copy number low, MSI-H = microsatellite instability-high, POLE = POLE mutation). The p values for two-by-two comparisons are shown. Differences were considered statistically significant when *p* < 0.05. **E** Bar charts representing differences in the frequency of different cell types in four molecular subtypes of endometrial cancer. (In the statistical analyses, ns represents no significant difference, * represents *p* ≤ 0.05, ** represents 0.05 < *p* ≤ 0.01, and *** represents 0.01 < *p* ≤ 0.001.) **F** Bubble diagram demonstrating differences in the composition of endometrial cancer cells of different molecular subtypes. Bubble plot in which the circle size represents the level of significance and the circle colour indicates which of the two comparisons on the y-axis has higher levels of the cell type on the x-axis

We examined 40 pathological white slices and selected 80 ROI points to ultimately capture 951,754 cells. UMAP visualisation shows the distribution of the subpopulations obtained from the dimensionality reduction clustering of all cells based on the Phenograph algorithm (Fig. 2A). Moreover, heatmaps were constructed to show the distribution of marker expression in different clusters (Fig. 2B). Ten cell subpopulations were defined by measuring the expression of different biomarkers on individual cells and merging cells with similar characteristics in the EEC ecosystem; they included T lymphocytes, B lymphocytes, natural killer (NK) cells, myeloid cells, endothelial cells, epithelial cells, fibroblasts, stromal cells, CD90 + cells and other lineages.

Myeloid cell have been subclassified through distinctive molecular markers. The myeloid population primarily comprises: granulocytes constitutively overexpressing S100A9, dendritic cells(DC) marked by CD11c, and functionally distinct macrophage subpopulations. Within the macrophage population, we further distinguished pro-inflammatory(M1) and anti-inflammatory (M2) phenotypes. The proliferating M1 subpopulation exhibited high Ki-67 expression and elevated CXCR4, a chemokine receptor closely associated with functional activation. In contrast, the M2 subpopulation characteristically expresses the scavenger receptor CD163, signifying its inhibitory functional state. Notably, CXCR4 expression levels exhibit significant variation across myeloid subpopulations, serving as a key indicator for distinguishing their functional activation states.

Within the T-lymphocyte compartment, we observed two important classical surface molecular markers, CD4 and CD8. Functionally activated CD4 + T cells, cytotoxic CD8 + T cells, FOXP3 + regulatory T cells (Tregs), and proliferating CD4 + T cells expressing both Ki67 and CXCR4 were efficiently identified. In particular, we note the important role played by CXCR4 molecules in T-cell biology. CXCR4 signalling promotes T-cell proliferation, potentially counteracting tumour-induced immunosuppression. In addition, high expression of CXCR4 on the surface of activated CD4 + T cells may lead to the production of cytokines that affect T-cell activation, thereby promoting the initiation of an effective anti-tumour response [18, 19]. Recent research has identified the role of the CXCL12-CXCR4 pathway in the lymphocytic infiltration of endometrial cancer [20]. It follows that the tumour microenvironment may alter CXCR4 expression on T cells in endometrial cancer [21]. Tumours can create a niche that modifies immune cell behaviour, including the expression of chemokine receptors.

Fibroblast metacellular clusters contain CD90 + fibroblasts, aSMA + myofibroblasts and inflammatory fibroblasts marked by SDF-1 + [22]. CD90 is a marker used to identify and isolate mesenchymal stem cells (MSCs), whereas SDF-1 is important for immune responses, especially during infections and inflammation [23, 24].

Furthermore, the clusters of endothelial cells are diverse and include CD90 + CD105 + endothelial cells, CD90-CD105- endothelial cells, and inflammatory and proliferating endothelial cells. In the context of immunology and cancer research, CD105 is thought to be a co-receptor for transforming growth factor β (TGFβ), which is involved in angiogenesis [25]. CD105 is commonly upregulated in activated endothelial cells and is particularly used as a marker of pathological proliferation, such as in tumours [25]. CD90, as a cell surface glycoprotein, is involved in cell adhesion, proliferation and differentiation and influences angiogenesis and interactions with immune cells [26]. CD90-CD105- endothelial cells could serve as a baseline for comparing pathological changes in the endothelial population within tumours. The differential expression of CD90 and CD105 on endothelial cells may provide valuable information on their functional role in the tumour microenvironment.

In our study, we performed a detailed analysis of cell distribution in patients with four different molecular subtypes of endometrial carcinoma, as shown in Fig. 2C, and found significant differences. After sample integration, each ROI averaged 11,896 cells. When the cell densities in each ROI region were combined, those in the POLE group were significantly greater than those in the CNL group (*P* = 0.033) and the MSI-H group (*P* = 0.005) (Supplementary Charting). Furthermore, the composition of cells from different molecular subtypes of endometrial cancer varies significantly, affecting the ecological structure and function of the tumour microenvironment (Fig. 2D). For example, compared with those in the CNL group, the proportions of active CD4 + T and cytotoxic CD8 + T-cell subpopulations in both the POLE group and the MSI-H group were significantly different (Fig. 2F). Intriguingly, the distribution characteristics of immunosuppressive Treg cells among the three molecular subtypes exhibited the same trend (Fig. 2E). With reference to the evidence of many recognised conclusions, the immunotherapy opinion of POLE-type and MSI-H-type endometrial cancers shows that the coordination of immune cell function in the complex immune microenvironment is important for maintaining microenvironmental homeostasis and is an important indicator of the efficiency of the immune response [27, 28].

Interestingly, the number of proliferating endothelial cells was greater in POLE-type and MSI-H-type endometrial cancers than in CNL-type endometrial cancers (Fig. 2F). The increased proliferative endothelial cell component in tumours with a good prognosis may seem counterintuitive at first, but several factors help explain this observation. An increase in proliferating endothelial cell components can indicate effective angiogenesis, a controlled growth environment, an enhanced immune response, and reduced hypoxia. These factors contribute to better patient outcomes by supporting more organised tumour behaviour and reducing aggressive characteristics. This pattern suggests that it is not the cell type alone but the dynamic balance of immune effector cells and proliferating endothelial cells that may be the key to achieving a positive therapeutic response. These findings emphasize the intricate interactions among different cellular components in shaping cancer therapy outcomes and highlight the need for personalized treatment strategies informed by comprehensive cellular profiling.

### Synchronized cellular mechanisms in the tumour landscape and correlation with clinical features

**Fig. 3:**
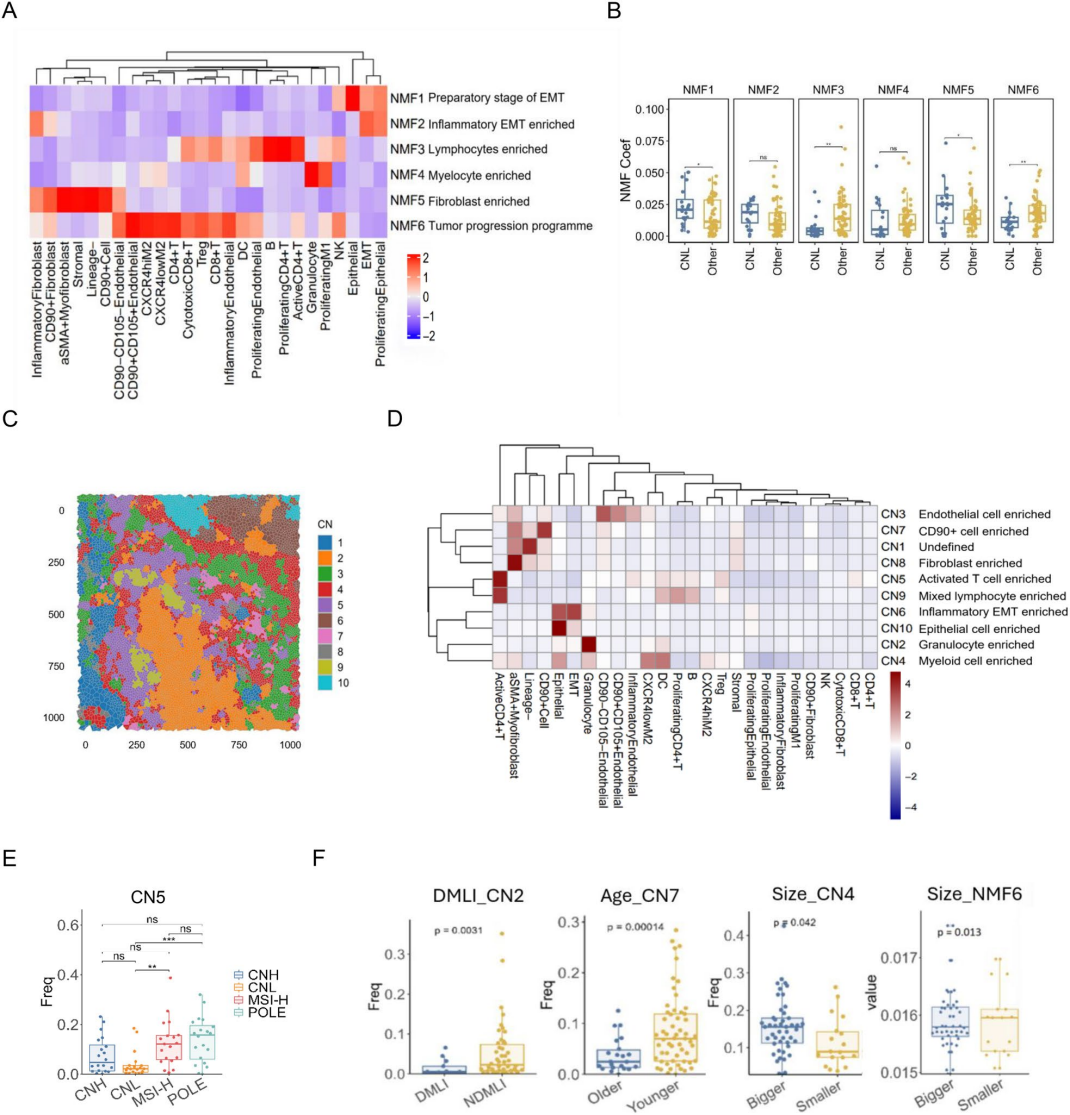
Synchronized cellular mechanisms in the tumour landscape and correlation with clinical features.** A** NMF-based cellular programs in the TME. The x-axis represents cell types in the TME, and the y-axis represents the loading of every cell type in each program defined in the table on the right. **B** Differences in the distribution of each NMF environment in CNL subtype compared with each of the other three molecular subtypes. **C** Schematic of the cellular neighbourhood (CN). **D** Heatmap of cell types represented across 10 CNs discovered in endometrial cancer. **E** Differences in the frequency of distribution of CN5 across the four molecular subtypes. **F** Correlations between ecological structure and clinical features in the tumour microenvironment. The upper left and upper right panels demonstrate the relationship between CN4 and NMF6 and tumour size, respectively, the lower left panel demonstrates the relationship between CN2 and tumour pathology with deep muscular infiltration, and the lower right panel demonstrates the relationship between CN7 and the age of onset of the patient. (In the statistical analyses, differences were considered statistically significant when *p* < 0.05; ns represents no significant difference, * represents *p* ≤ 0.05, ** represents 0.05 < *p* ≤ 0.01, and *** represents 0.01 < *p* ≤ 0.001.)

We utilized a non-negative matrix factorization (NMF)-based approach to capture the coordinated cellular programs of fine-grained cellular subpopulations within the tumour microenvironment (TME) and to investigate the differences in the intra-tumoural environment across the four molecular subtypes. From the tumour samples, we identified six cellular programs (NMF1-6) (Fig. 3A). NMF1 is characterized by epithelial and proliferating epithelial cells, representing the preparatory stage of epithelial-mesenchymal transition. NMF2 consists of inflammatory epithelial-mesenchymal transition (EMT) cells and inflammatory fibroblasts, reflecting the outcome of epithelial-mesenchymal transformation. NMF3 is predominantly enriched in various lymphocyte types, including activated CD4 + T cells, CD8 + T cells and B cells. NMF4 is primarily composed of granulocytes, proliferating M1 cells, and dendritic cell types. In this context, dendritic cells function as antigen-presenting cells, stimulating T-cell responses and enhancing immune system activation. This activation is further amplified by neutrophils and contributes to the establishment of an inflammatory environment alongside the aforementioned cell types, promoting anti-tumor activity through the release of cytokines and chemokines that recruit additional immune cells to the tumour site. NMF5 consists of fibroblasts that form the mesenchymal environment, whereas NMF6 encompasses a diverse array of enriched cell types, including CD90 + CD105 + endothelial cells, M2 macrophages and regulatory T cells (Tregs). This program is described as a “tumour progression programme," which may represent synergy in the creation of a supportive tumour niche.

In the present study, we identified notable cellular infiltration and ecological features of CNL-type tumours through NMF cellular program analysis. Interpreting these phenomena will not only benefit patients with CNL-type endometrial cancer but also enhance our overall understanding of tumour biology and treatment strategies for gynaecological malignancies. Compared with the other three molecular subtypes, CNL-type tumours exhibited higher percentages of NMF1 and NMF5 and lower percentages of NMF3 and NMF6 (Fig. 3B). Additionally, CNL-type endometrial cancer is characterized by a marked increase in epithelial-mesenchymal transition (EMT), which enhances the motility and invasiveness of tumour cells. Concurrently, decreased lymphocyte infiltration limits the tumour’s ability to mount an effective immune response, creating a synergistic environment that favours tumour growth and progression.

Given these findings, tailored therapeutic strategies are urgently needed. For instance, targeting pathways involved in the EMT process, such as growth factor signal transduction, and modifying the immunosuppressive characteristics of the tumour microenvironment may mobilize a more effective immune response. Furthermore, identifying specific biomarkers associated with EMT and immune infiltration in CNL-type tumours could improve prognostic assessments and inform adjuvant therapy decisions. This approach aims to facilitate a more personalised management strategy that considers the unique characteristics of each tumour.

NMF offers a robust framework for identifying and characterising cellular neighbourhoods within the tumour microenvironment. We next explored whether multicellular structures within tumours would yield valuable insights into the dynamics of the EEC tumour microenvironment (TME). To evaluate this, we first identified the twenty nearest spatial neighbours around each individual cell, referred to as cellular neighbours (CNs). By mapping the 10 CNs with different colours in different ROIs, the spatial distribution patterns of different CNs can be displayed as shown in Fig. 3C. A k-means clustering method (k = 10) was subsequently applied to reclassify and annotate these neighbours based on their cellular components. The resulting clusters were designated as follows: undefined (CN1), granulocyte enriched (CN2), endothelial cell enriched (CN3), myeloid cell enriched (CN4), activated T cell enriched (CN5), inflammatory EMT enriched (CN6), CD90 + cell enriched (CN7), fibroblast enriched (CN8), mixed lymphocyte enriched (CN9), and epithelial cell enriched (CN10) (Fig. 3D). When the distribution characteristics of cellular neighbourhoods across the four molecular subtypes were compared, the frequency of CN5 was significantly greater in the POLE and MSI-H types than in the CNL type (Fig. 3E). These findings underscore the critical importance of activated T-cell enrichment for endometrial cancer prognosis. In addition to its correlation with survival, we identified other relationships between ecological structure and specific clinical subpopulations. For instance, the CD90 + cell subpopulation demonstrated age-based enrichment, while myeloid cell enrichment and NMF6 expression were associated with tumour size (diameter > 2 cm). Moreover, patients with granulocyte-enriched tumours were less likely to exhibit deep muscle infiltration (Fig. 3F).

### Dynamic cell distribution in the epithelial, mesenchymal, and immune regions of the endometrial cancer ecosystem across molecular subtypes

**Fig. 4:**
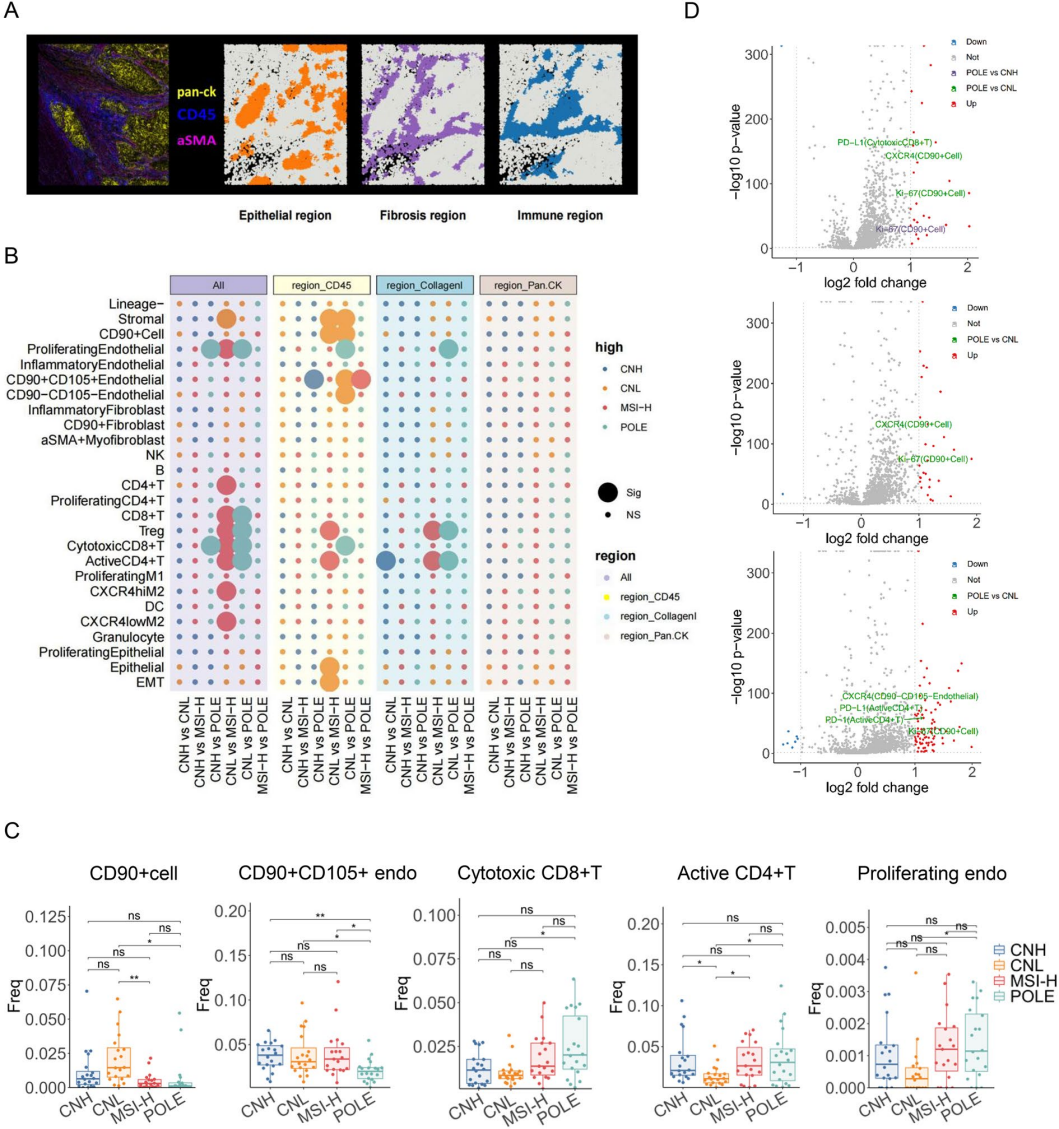
Dynamic cell distribution in the regions across molecular subtypes. **A** This panel presents a detailed schematic illustration of the tumour microenvironment (TME) regions, including epithelial, immune, and fibrotic zones. The illustration is enhanced with an imaging mass cytometry (IMC) image overlay, visually delineating the boundaries and characteristics of each region, thereby facilitating a clearer understanding of the spatial analysis methodology. **B** The panel quantifies the cellular composition within various tumour microenvironment (TME) regions and compares the proportions of major immune and stromal cell types across different molecular subtypes. The analysis emphasizes the identification of significant changes associated with molecular subtypes, as well as variations across functional regions and overall composition. **C** This statistical plot presents a comparative analysis of cell distribution across different regions of the tumour microenvironment (TME). The results for various cell frequencies within the four molecular subtypes are displayed. The top three subplots illustrate the cell distribution in the immune region, whereas the bottom two subplots show comparative results in the fibrotic region. (ns represents no significant difference, * represents *p* ≤ 0.05, ** represents 0.05 < *p* ≤ 0.01, and *** represents 0.01 < *p* ≤ 0.001.). **D** Functional marker analysis across the TME region. The volcano plot shows changes in functional markers, including immune checkpoints and inflammatory markers, within the TME region. It presents the expression of each two of the four molecular subtypes within three regions, highlighting the dynamics of the characterised molecules. Each point represents the average fold change for a specific marker in a cell type in a given comparison group, with the x-axis representing the log2-fold change and the y-axis representing the -log10 p value

Previous studies have shown that complex cellular functional specialisations cause these regions to exhibit different interactions and responses to therapy [29]. Therefore, to fully understand the possible heterogeneity of spatial features within the tumour, we explored the TME comprising different regions, such as epithelial, immune and fibrotic regions (Fig. 4A). In terms of the immune region, the enrichment of immune cells in the four molecular subtypes of endometrial carcinomas revealed some unusual findings compared with previous overall tumour results (Fig. 4B, C). Accompanying the decrease in cytotoxic CD8 + T cells and proliferating endothelial cells in the POLE type was an increase in CD90 + CD105 + endothelial cells in the CNH type. Furthermore, compared with the CNL-type progressive increase in CD90 cells and epithelial mesenchymal transition, the MSI-H subtype had a decrease in proliferating endothelial cells. Additionally, the immunological regions differed from the overall cellular distribution in that both the CNL-type and MSI-H-type had more CD90 + CD105 + endothelial cells than the POLE-type, whereas the CNL-type had more stromal cells.

In the fibrous region, significantly fewer active CD4 + T cells were observed in the CNL type, and fewer proliferating endothelial cells and cytotoxic CD8 + T cells were observed in the POLE type than in the CNH type. However, within the epithelial region, none of the four molecular subtypes of endometrial cancer were significantly different.

Having established the variation in cellular composition across distinct regions of the tumour microenvironment, it is essential to delve deeper into the functional implications of these differences. The frequency of specific cell types not only influences overall cellular dynamics but is also correlated with the expression profiles of key surface markers that govern cellular interactions and behaviour. Thus, the following analysis focuses on the differential expression of surface marker molecules, highlighting their relevance in shaping the interactions and functions of the diverse cell populations present within the tumour microenvironment. Across all regions, the proliferation marker Ki-67 and the activation molecule CXCR4 were significantly elevated in CD90 + cells of the POLE subtype (Fig. 4D). Furthermore, compared with the CNL subtype, the POLE subtype, which exhibited the most favourable prognostic performance, demonstrated significantly greater expression of CXCR4 on CD90 − CD105 − endothelial cells in the epithelial region. CXCR4-expressing CD90 + cells demonstrated enhanced migration towards sites of inflammation, tissue injury, or tumours, facilitating T-cell recruitment and activation and thereby promoting tissue repair, regeneration, and antitumor immunity. Furthermore, in the epithelial region, the expression levels of CXCR4 on CD90 − CD105 − endothelial cells—characterized by their role in normal vascular function and homeostasis, without invasive tumour-associated characteristics—were significantly greater in the POLE subtype than in the CNL subtype of endometrial cancer. This synergistic immunomodulation and tissue repair may contribute to the favourable prognosis associated with the POLE subtype [30]. However, PD-L1 was significantly overexpressed on CD8 + T cells in the POLE subtype compared with those in the CNL molecular subtype. These findings may clarify the paradoxical biological aggressiveness of the POLE subtype, despite its generally favourable prognosis. Elevated PD-L1 expression on effector T cells can promote an immune-tolerant tumour microenvironment through interactions with neighbouring antigen-presenting cells and functional T cells [31, 32].

### *Focus on the distribution and tumour promotion of CD90* + *CD105* + *endothelial cell populations within immune regions*

Consistent with previous studies, our study revealed significant differences in the cellular composition, spatial distribution and molecular characteristics of different functional regions of endometrial cancer, including immune microenvironment-enriched, epithelial differentiated and fibrotic mesenchymal regions.

**Fig. 5:**
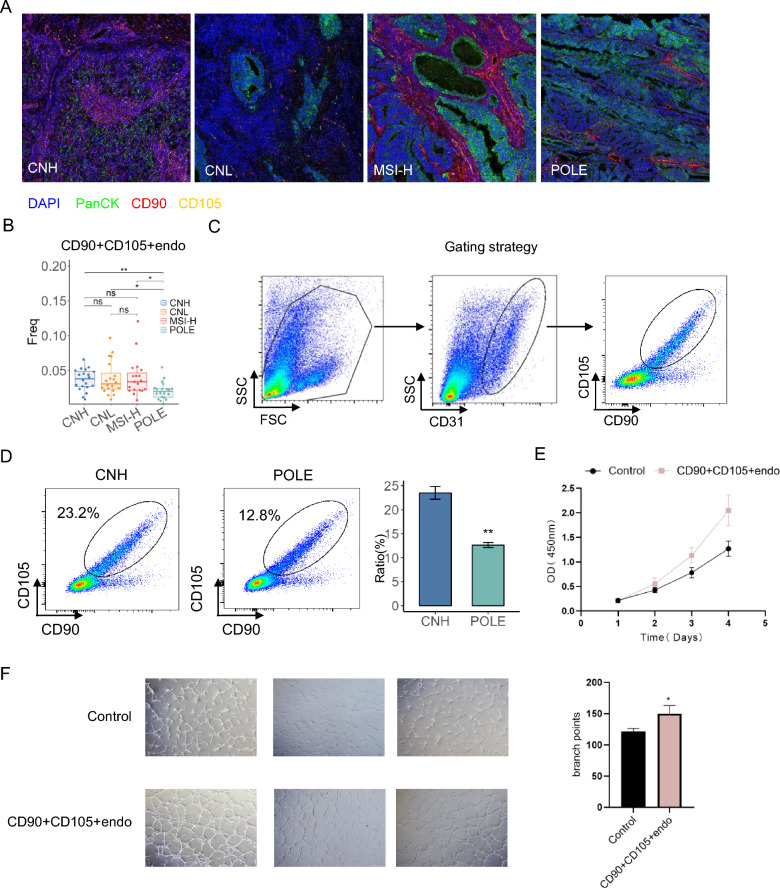
Differential distribution and tumour promotion of CD90 + CD105 + cells in immune regions.** A** IMC labelling of CD90 and CD105 double-positive cells in four molecular subtypes of endometrial cancer. DAPI stains the nucleus blue, PanCK stains the epithelium green, CD90 labels the red region, and CD105 labels the yellow region. **B** Histogram visualisation showing the frequency statistics of CD90 and CD105 double-positive cells in the four molecular subtypes. (ns represents no significant difference, * represents *p* ≤ 0.05, ** represents 0.05 < *p* ≤ 0.01, and *** represents 0.01 < *p* ≤ 0.001.). **C** Representative flow cytogram demonstrating a gating strategy for determining CD90 and CD105 double-positive endothelial cells from ten patients. **D** Quantification of CD90 + CD105 + endothelial cells in patients with endometrial cancer of the CNH and POLE subtypes. ** *P* < 0.01. *p* values were determined using the Wilcoxon rank-sum test. **E** CCK-8 assay to detect the effect of CD90 + CD105 + endothelial cells on the proliferative capacity of endometrial cancer cells. After co-culture of CD90 + CD105 + endothelial cells selected by flow sorting with the endometrial cancer cell line Ishikawa, the tumour proliferative capacity was detected for 4 consecutive days, and growth curves were plotted. **F** The angiogenesis experiments were divided into two groups: a CD90 + CD105 + endothelial cell experimental group and a control group without surface molecular markers. The experimental results showed that CD90 + CD105 + endothelial cells promoted tumour angiogenesis and thus accelerated the proliferation of endometrial cancer cells compared with that of the control group. * represents *p* ≤ 0.05

First, we systematically analysed the distribution of CD90 + CD105 + endothelial cell populations across the four molecular subtypes of endometrial cancer (POLE, MSI-H, CNH and CNL) utilizing imaging mass cytometry (IMC) technology (Fig. 5A). Notably, the POLE subtype exhibited a significantly sparse distribution of these cells, in contrast to the other three subtypes, which displayed a high enrichment of CD90 + CD105 + endothelial cells. Quantitative analysis further confirmed that the proportion of CD90 + CD105 + endothelial cells in the POLE subgroup was significantly lower than that in the CNH (*P* < 0.01), MSI-H (*P* < 0.05) and CNL subtypes (*P* < 0.05) (Fig. 5B). Moreover, we validated the differential distribution of this double-positive endothelial cell population within the immune regions of the various subtypes using flow cytometry (Fig. 5C). Based on the prognostic value of the molecular subtypes of endometrial cancer, we chose the POLE subtype and the CNH subtype, whose prognostic value is the best and the worst, respectively, and is not controversial, to analyse specifically the distribution of these groups of cells. Our findings revealed that compared with the POLE subtype, the CNH subtype contained a significantly greater percentage of CD90 + CD105 + endothelial cells (23.2%) (12.8%, *P* < 0.05) (Fig. 5D). Moreover, CD90 + CD105 + endothelial cells were sorted by flow cytometry and co-cultured with Ishikawa cells, and this particular group of cells significantly accelerated endometrial cancer proliferation (Fig. 5E). Considering the stemness markers on the surface of this population of endothelial cells, we also experimentally verified their role in promoting angiogenesis, which may play an important role in shaping the tumour microenvironment (Fig. 5F).

The specific enrichment of this cell population in the high-risk CNH subtype highlights a promising avenue for future research focused on prognostic assessment and the development of targeted therapies. This enrichment may inform strategies that combine anti-angiogenic and inhibition of proliferative metabolism approaches, warranting further investigation into the underlying molecular mechanisms.

### Pairwise cellular spatial interactions and survival outcome in endometrial cancer ecosystem across molecular subtypes

**Fig. 6:**
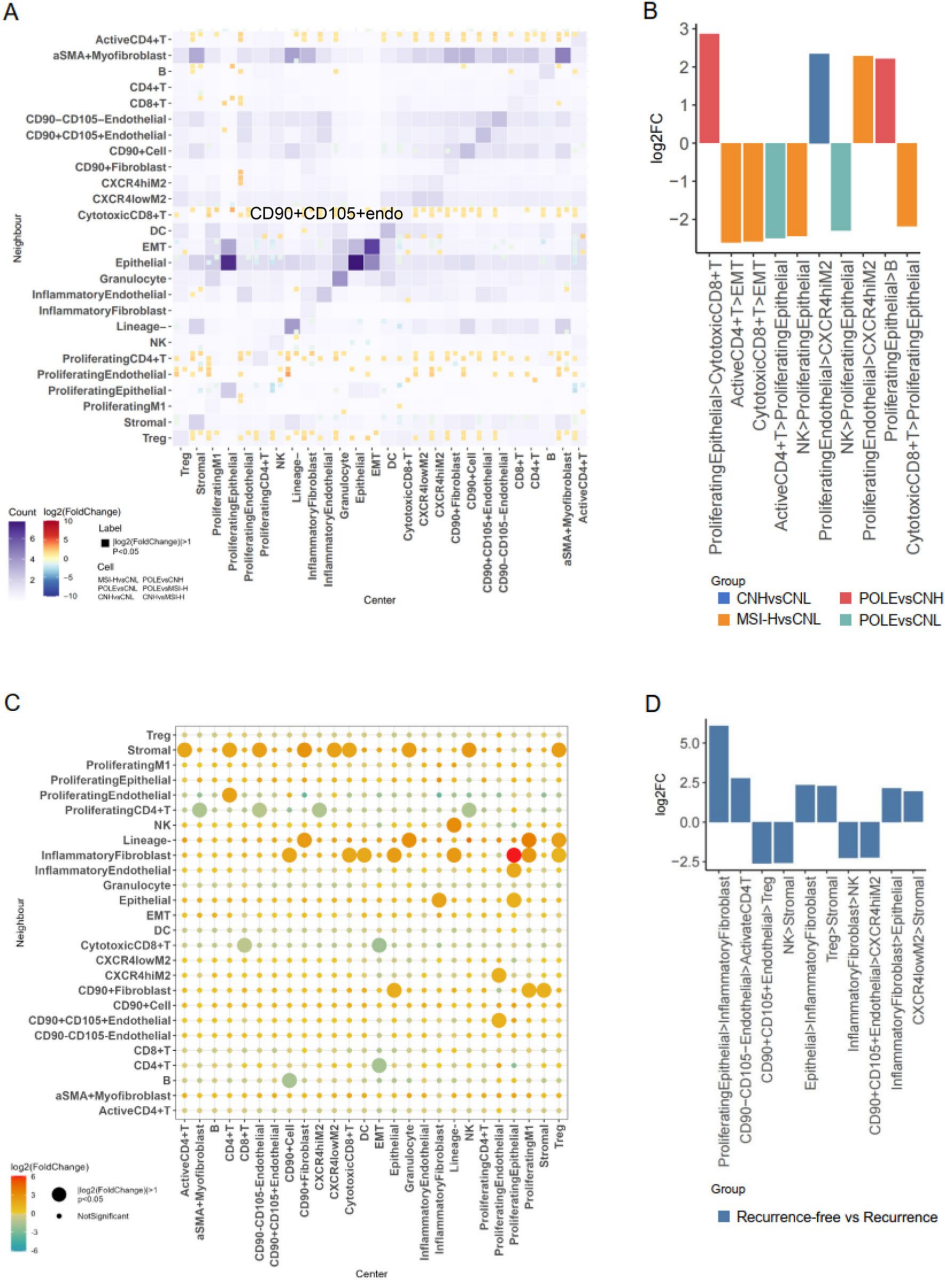
Pairwise cellular spatial interactions and survival outcomes in endometrial cancer ecosystems across molecular subtypes.** A** The figure shows the pattern of cell–cell interactions. Each tile represents the log fold change in the corresponding cell–cell interaction, and only comparisons with t test p values less than 0.05 are shown in the figure. **B** This figure illustrates cell–cell interactions with the highest differential significance within tumour ecological structures, characterizing each of the four molecular subtypes. The height of the bars represents the log fold change in interactions between the comparison groups. **C** Interaction patterns in immune regions. This figure demonstrates the pattern of cell–cell interactions within the immune regions of CNH-type patients with different prognostic outcomes, highlighting the tumour microenvironment of highly aggressive types of tumours. Each tile represents the log2 fold change in the respective cell–cell interactions, and only comparisons with t test *p* values < 0.05 are shown on the plot. “0” indicates the good prognosis group, and “1” indicates the recurrence group. **D** Cell–cell interactions with the most pronounced differences within immune regions. This figure shows the difference in the pattern of cellular interactions between the good prognosis and relapse groups, further emphasising the importance of spatial cellular interactions in mediating a positive clinical outcome. The height of the bars corresponds to the log fold change in interactions between the two groups: 0 indicates the good prognosis group, and 1 indicates the recurrence group

To refine our overall understanding of the EEC ecosystem beyond cell frequency, we next explored the interactions between cellular phenotypes. We extracted all the microenvironmental cell populations in our dataset (including endothelial cells, myeloid cells, or lymphocytes) and compared the differences in cellular interactions for each of the two molecular subtypes separately (Fig. 6A). POLE-type CD90 + cells significantly interact with both proliferating endothelial cells and effector T cells. In contrast, MSI-H-type stromal cells clearly interacted with proliferative CD4 + T cells, whereas CNL-type cells displayed stronger interactions between immune effector cells (including proliferative CD4 + T cells and NK cells) and epithelial-mesenchymal transition cell types.

Combining the results of all two-by-two comparisons among molecular subtype groups, we identified the top ten most significant differences in cellular interactions, as illustrated in Fig. 6B. We focused on characterizing the intercellular interaction patterns of CNL subtypes, which remain poorly defined. Our analysis revealed significant interactions between activated CD4 + T cells, cytotoxic CD8 + T cells, and epithelial-mesenchymal transition (EMT) cells in CNL subtypes. Additionally, interactions between NK cells and proliferating epithelial cells were notably significant in the CNL subtype compared with those of the MSI-H and POLE subtypes, which are associated with better prognosis. Prognostic analyses revealed that the interactions between proliferating endothelial cells and M2 macrophages with high CXCR4 expression in the CNH subtype, characterized by the worst prognosis, are critical. Conversely, the most significant interactions in the POLE subtype, associated with the best prognosis, involved proliferating epithelial cells and immune cells, including cytotoxic CD8 + T cells and B cells.

We then focused our study on patients with CNH subtypes to investigate differences in cellular spatial interaction patterns within immune regions among individuals with varying prognoses for highly aggressive tumours (Fig. 6C). The top ten cellular interaction patterns exhibiting the most significant differential features are detailed in Fig. 6D. Notably, interactions between CD90 + CD105 + endothelial cells and the immunosuppressive environment (comprising M2 macrophages with high CXCR4 expression and Tregs) strongly predicted poor outcomes, whereas interactions between CD90 − CD105 − endothelial cells and activated CD4 + T cells resulted in a more favourable prognosis. Additionally, the complex interactions between NK cells and mesenchymal cells may indicate a poorer outcome than the interactions between inflammatory fibroblasts and proliferating epithelial cells.

### *Focus on the immunoregulatory function of CD90* + *CD105* + *endothelial cells in the tumour microenvironment*

In the above algorithmic model, the CD90 + CD105 + endothelial cell-macrophage interactions significantly contributed to the prediction of recurrence in endometrial cancer patients. Therefore, we arranged experiments to further validate the reciprocal patterns of the two cell types and to better understand the complex relationships in the endometrial cancer tumour microenvironment.

**Fig. 7:**
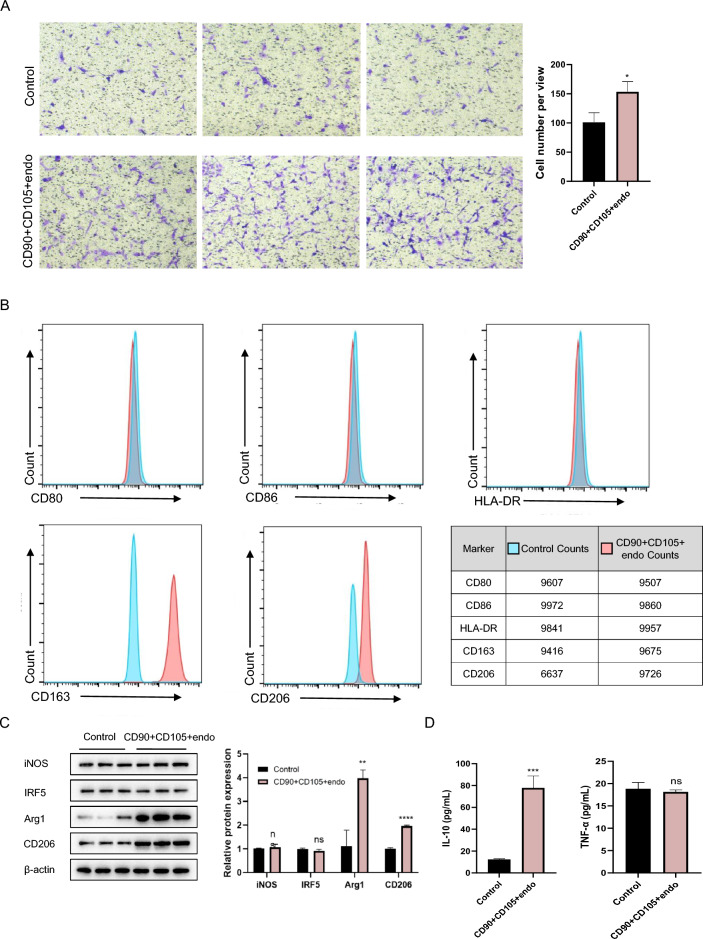
Immunoregulatory function of CD90 + CD105 + endothelial cells. **A** Transwell migration assays in which endothelial cells were inoculated in the lower compartment and macrophages were inoculated in the upper compartment. CD90 + CD105 + endothelial cells could recruit more macrophages. * represents *p* ≤ 0.05. **B** CD90 + CD105 + endothelial cells isolated from tumour samples were co-cultured with THP1 cells, and macrophage polarisation was detected by flow cytometry. CD80, CD86, and HLA-DR are markers of M1-type cellular polarisation, and CD163 and CD206 are molecular markers of M2-type cells. **C** CD90 + CD105 + endothelial cells were co-cultured with THP1 cells, and macrophage polarisation was detected by Western blot protein immunoblotting. iNOS and IRF5 are M1-type macrophage markers, and Arg1 and CD206 are M2-type cell markers. **D** CD90 + CD105 + endothelial cells were co-cultured with THP1 cells, and cytokines were detected by ELISA. TNF-αis an M1-associated cytokine, and IL-10 is an M2-associated cytokine. (In the statistical analyses, differences were considered to be statistically significant when *p* < 0.05; ns represents no significant difference, * represents *p* ≤ 0.05, ** represents 0.05 < *p* ≤ 0.01, *** represents 0.01 < *p* ≤ 0.001, and **** represents 0.001 < *p* ≤ 0.0001.)

Transwell experiments in which CD90 + CD105 + endothelial cells were inoculated in the lower chamber and macrophages were cultured in the upper layer revealed that the number of macrophages migrating to the lower layer increased, demonstrating that CD90 + CD105 + endothelial cells play a role in macrophage recruitment (Fig. 7A). Moreover, CD90 + CD105 + endothelial cells obtained from sorted human tumour samples were co-cultured with THP1 cells, and flow cytometry analysis revealed that the expression of M1 markers was low or almost non-expressed, whereas the expression of M2 markers was significantly high in the co-cultured group (Fig. 7B). Similarly, Western blot experiments to detect protein expression found that M1 markers were almost unchanged and M2 markers were significantly highly expressed (Fig. 7C). In addition, the results of the ELISA-based cytokine assays revealed no significant difference in the expression of TNF-α, an M1-related cytokine, in the CD90 + CD105 + endothelial cell co-culture group, but the expression of IL-10, an M2-related cytokine, was significantly upregulated compared with that in the control group (Fig. 7D).

Combining the results of the IMC analysis and cellular experiments, the immunoregulatory role of CD90 + CD105 + endothelial cells in tumour ecology should not be ignored. This population of cells participates in the macrophage M2 polarization process, with which they co-shape the tumour-suppressive immune microenvironment, promoting malignant phenotypes and increasing tumour resistance.

### Identifying spatial features of four molecular subtypes and the ecological structure of tumours with poor prognosis for CNH subtypes using machine learning

**Fig. 8:**
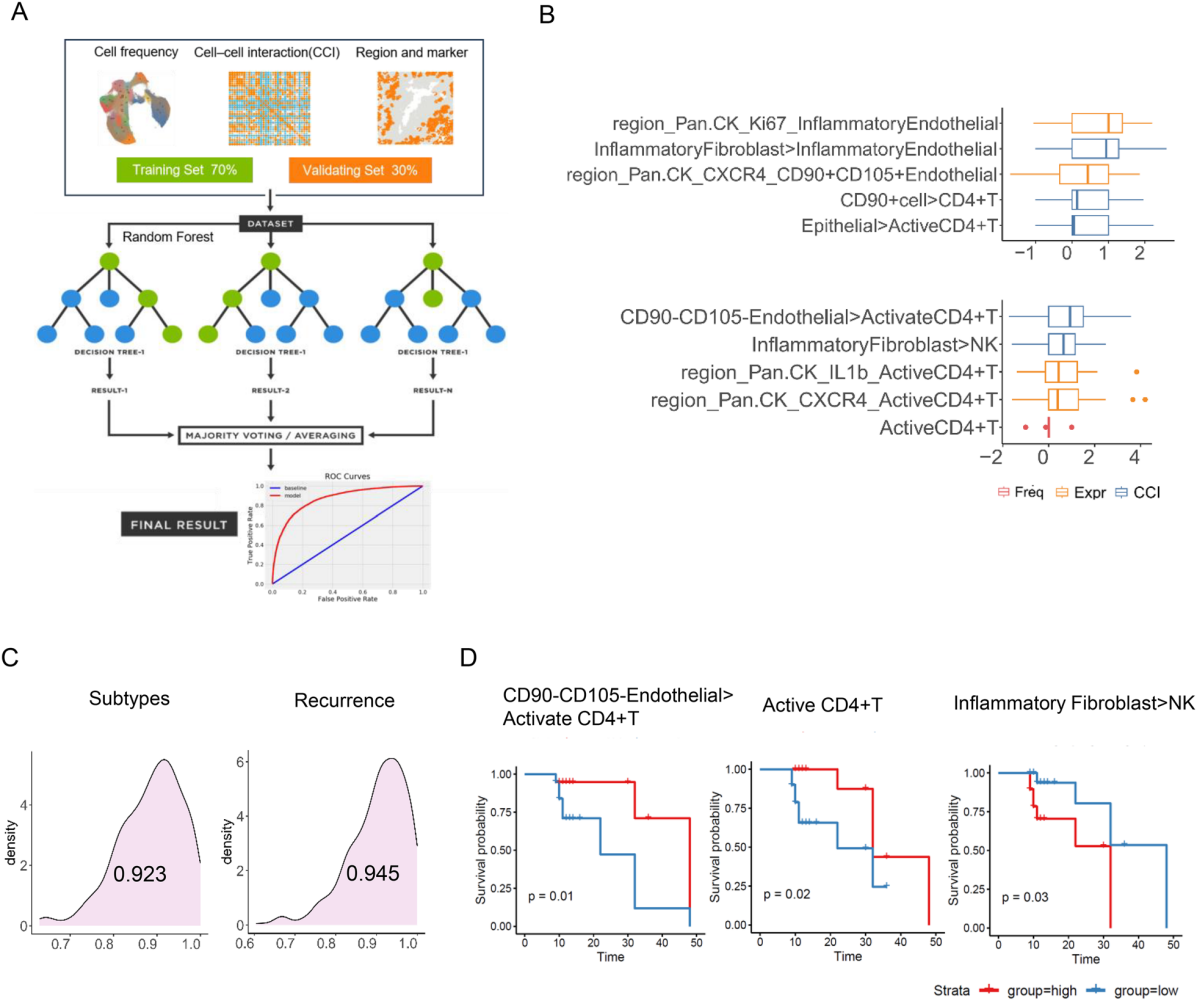
Identifying spatial features of four molecular subtypes and the ecological structure of tumours with poor prognosis for CNH subtypes using machine learning. **A** Schematic diagram of a random forest analysis that shows the easy flow and science of the machine learning algorithm. **B** Spatial characteristics of the four molecular subtypes (above) and prognostically relevant ecological patterns of CNH invasive subtypes (below). This box plot shows the key factors for predicting molecular subtypes and prognosis identified by random forest analysis. The most important features are highlighted, including the expression of IL-1B and CXCR4 on activated CD4 + T cells and specific TME regions on activated CD4 + T cells. These expression levels were predominantly increased in the good prognosis group. This visual display emphasises the predictive value of these functional molecular markers in predicting prognosis. **C** AUC plots and average values after 1000 repetitions of calculations for two random forest models. **D** K-M one-way survival analysis. With respect to the spatial characteristics and prognostic associations of CNH invasive subtypes, statistical analyses revealed that CD90-CD105-endothelial and activated CD4 + T-cell interaction patterns as well as the frequency of active CD4 + T cells were desirable factors favouring tumour repair; however, inflammatory fibroblast and NK cell interactions were prognostic independent risk factors

Given the complexity of the tumour microenvironment (TME), we analysed a comprehensive dataset that included cell population frequencies, regional characteristics, cellular interactions, and expression profiles of key functional markers. These features were assessed using a random forest (RF) model to determine their importance in predicting treatment outcomes (Fig. 8A). Ultimately, the top five features were selected to construct a tumour spatial microenvironment model based on the molecular subtype and prognosis of CNH-invasive tumours (Fig. 8B). The random forest model analysis of these five features yielded a receiver operating characteristic (ROC) curve of 0.923 for the molecular subtype and 0.945 for the recurrence model (Fig. 8C). Consistent with the Kaplan–Meier univariate analysis, IL-1β expression on activated CD4 + T cells was found to be crucial for enhancing immune activation, promoting cytokine production, and facilitating interactions with other immune cells. Additionally, interactions between CD90 − CD105 − endothelial cells and activated CD4 + T cells were identified as critical for repairing the tumour ecological structure. In contrast, the interaction pattern between inflammatory fibroblasts and NK cells emerged as an independent risk factor for tumour prognosis (Fig. 8D).

## Discussion

In this study, we employed a single-cell proteomics approach to investigate the characteristics of the tumour microenvironment (TME) across four molecular subtypes of endometrial cancer. By focusing on the frequency of cell types and patterns of cellular interactions, we aimed to provide a comprehensive understanding of how these dynamics contribute to tumour behaviour and progression.

Our analysis revealed distinct patterns of cellular composition among the molecular subtypes. The variation in the frequency of specific immune and stromal cell types highlights the unique biological and pathological features associated with each subtype. For instance, CNL-type tumours exhibit a higher proportion of epithelial and fibroblastic cells than the other subtypes, which correlates with active epithelial-mesenchymal transition. In contrast, the POLE and MSI-H subtypes demonstrate increased immune cell infiltration, indicating a more robust immune response. These findings underscore the importance of evaluating not only the presence of individual cell types but also their relative abundance within the tumour microenvironment (TME). Such differential cellular compositions can significantly influence tumour progression, therapeutic responses, and overall patient outcomes.

In addition to examining the frequency of cell types, our study emphasises the significance of cellular interactions within the tumour microenvironment (TME). We investigated not only pairwise interactions but also complex relationships within cellular neighbourhoods (CNs) and NMF cellular programming units. This holistic perspective is crucial for understanding the cooperative and competitive dynamics that drive tumour behaviour. For instance, we observed that spatial interactions between CD90 + cells and immune effector cells, such as CD8 + T cells, activated CD4 + T cells, and regulatory T cells, varied significantly across molecular subtypes, particularly outside immune regions. Within immune regions, the dynamic trends of CD90 + cells and CD90 + CD105 + endothelial cells also differed among the subtypes. These observations underscore the critical role of CD90 as a stemness marker in mesenchymal stem cells (MSCs), suggesting that varying differentiation tendencies may influence tumour biological behaviours [33, 34]. Moreover, the prognostic value of CD90 + CD105 + endothelial cells was fully validated in this study. A spatial proteomics study revealed that not only the enrichment of CD90 + CD105 + cells but also their pattern of interactions with immunosuppressive cells, including M2 cells and Tregs, in the immune system were associated with poor prognosis [35–37]. Similarly, cellular experiments confirmed that this population of cells could chemotax immunosuppressive cells and promote angiogenesis to construct an immunosuppressive microenvironment. Consequently, the spatial and temporal distribution of the CD90 + CD105 + endothelial cell population is essential for shaping the ecological structure of tumours.

By integrating all spatial proteomic features within the endometrial cancer tumour ecosystem, we successfully modelled the microenvironments of the four molecular subtypes, achieving a commendable model fit with an area under the curve (AUC) of 0.923. Furthermore, we developed a recurrence prediction model specifically for the CNH molecular subtype of highly aggressive endometrial cancers, incorporating the top five spatial proteomic features, which yielded an AUC as high as 0.945. This model enables differentiation between various cellular components and interaction patterns within aggressive tumours that share the same molecular signature [38]. These findings highlight the potential of single-cell measurements to bridge the existing gap in understanding the heterogeneous response states of patients with identical molecular subtypes of endometrial cancer.

Ultimately, these insights not only enhance our understanding of endometrial cancer biology but also inform the development of more effective personalized treatment strategies. Patients can be stratified using functional biomarkers associated with specific cellular interactions and immune profiles, allowing for tailored treatment adjustments that could improve clinical outcomes.

Our study has significant implications for the development of targeted therapeutic strategies. Identifying specific cellular neighbourhoods and their functional roles can guide the design of combination therapies that enhance the immune response while simultaneously addressing the invasive properties of tumours. For example, strategies aimed at inhibiting EMT pathways may be particularly beneficial in CNL-type tumours, especially when combined with immunotherapies designed to increase lymphocyte infiltration. Additionally, interventions targeting the differentiation of CD90 + cells CD90 + CD105 + endothelial cell production may further enhance the efficacy of the immune response.

While our study provides valuable insights into the TME of endometrial cancer, the following limitations must be recognised. The current analysis is primarily based on cross-sectional data, which, while revealing significant associations between cellular subpopulations and clinical characteristics and outcomes, lack sufficient validation of causal relationships. Future studies should employ organoid co-culture experiments or humanised mouse models for functional validation and conduct multi-time point sampling cohort studies to track the dynamic evolution of the TME. Furthermore, this study focuses on proteomic-level characteristics, but the regulation of the tumour microenvironment involves multi-level interactions such as genomic variations, epigenetic modifications, and cellular metabolic reprogramming [39]. Subsequent studies integrating spatial transcriptomics and multiplex immunofluorescence techniques can establish molecular network associations while preserving spatial information. In addition, there is still room for improvement in the technical aspects of this study, such as developing mass spectrometry flow cytometry panels with higher parameters, as more markers can enhance the resolution of clustering algorithms. Finally, and most importantly, the clinical translation pathway remains a challenge. Although this study demonstrated the target roles of CD90, CD105, and CXCR4, their clinical applicability still needs to be validated in prospective clinical trials.

Addressing these limitations will depend on interdisciplinary collaboration, leveraging multiomics analysis of pre- and post-treatment paired samples to assess the efficacy of biomarker-guided dynamic treatment adjustments. The ultimate goal is to establish an iteratively optimised “digital TME twin” platform to provide a real-time prediction model for individualised treatment decisions.

## Conclusion

This study elucidates the heterogeneity of the tumour microenvironment across four molecular subtypes of endometrial carcinoma, emphasising the roles of cellular interactions and synchronised cellular programmes. Furthermore, it identifies a distinct population of cells, CD90 + CD105 + endothelial cells, which participate in establishing an immunosuppressive microenvironment by influencing macrophage polarisation, thereby indicating poor prognosis. By extracting molecular and cellular information from the tumour ecological microenvironment, characteristic models identifying the four molecular subtypes and a prognostic risk prediction model for high-risk infiltrative tumours were constructed respectively. These hold reference value for clinical treatment decision-making.

## Supplementary Information

Additional file1

Additional file2

## Data Availability

Data that support the findings of this study have been deposited in the website (https://ngdc.cncb.ac.cn/omix/preview/Mx97OqP1). Additionally, western blotting data is provided within the supplementary information.

## Abbreviations

EEC: Endometrioid endometrial carcinoma
ASMR: Age-standardized mortality rate
POLE: Polymerase epsilon
CNL: Copy-number low
CNH: Copy-number high
MSI-H: Microsatellite instability-high
TME: Tumour microenvironment
IMC: Imaging mass spectrometry
FFPE formalin-fixed: Paraffin-embedded
CN: Cell neighbourhood
NMF: Non-negative matrix factorization
EMT: Epithelial-mesenchymal transition
RF: Random forest
MSCs: Mesenchymal stem cells
