# Supplementary figures and images for: Spatial ecostructural modelling of endometrial cancer identifies the key role of CD90 + CD105 + endothelial cells in tumour heterogeneity and predicts disease recurrence
Source: Exp Hematol Oncol. 2025 Nov 17;14:132. doi: 10.1186/s40164-025-00724-6 (PMC12621410; doi:10.1186/s40164-025-00724-6)

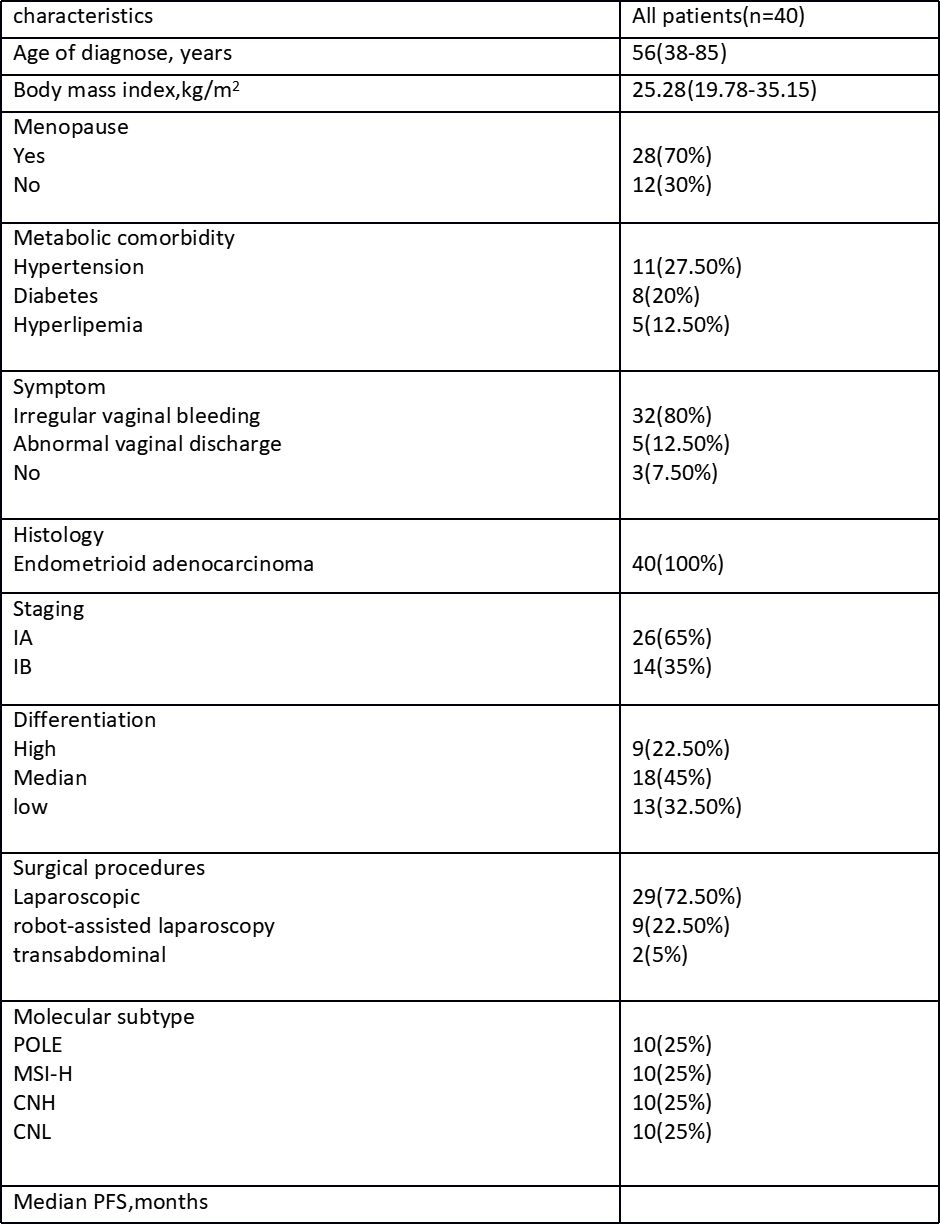


Supplementary Table

clinical and pathological characteristics in the enrolled patient

Supplement: Supplementary file 1 — Additional file1 [file 40164_2025_724_MOESM1_ESM.docx]

1.actin


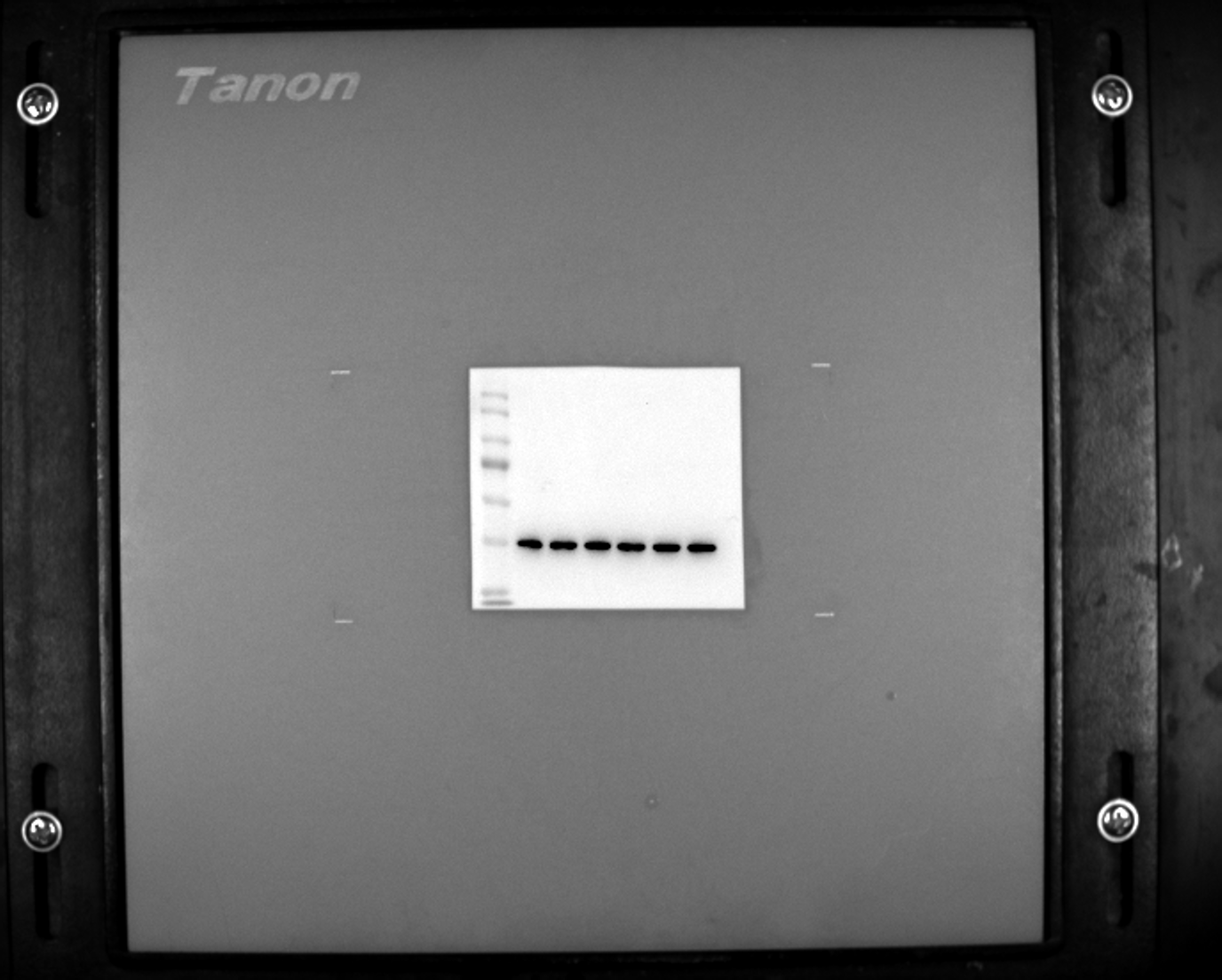

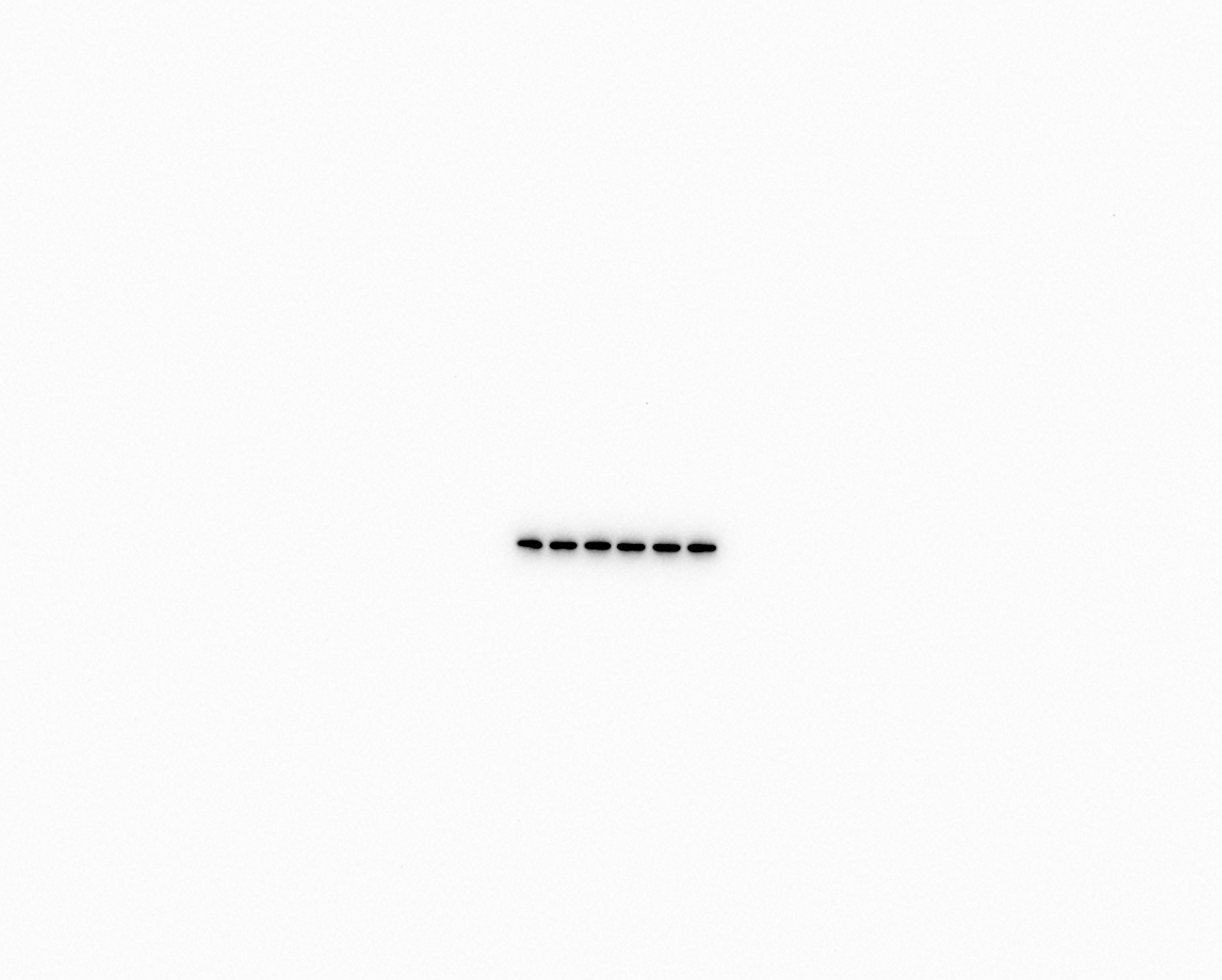

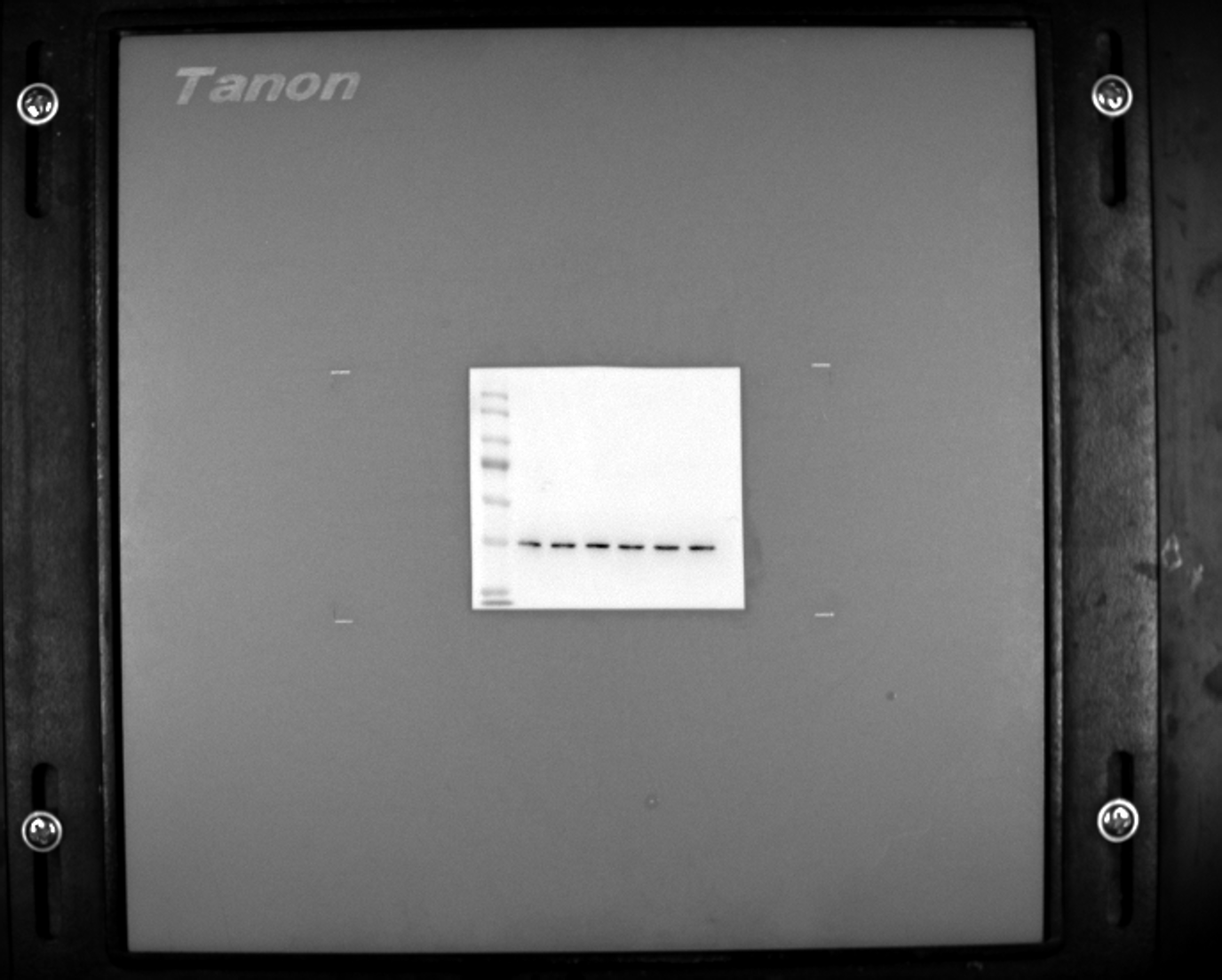

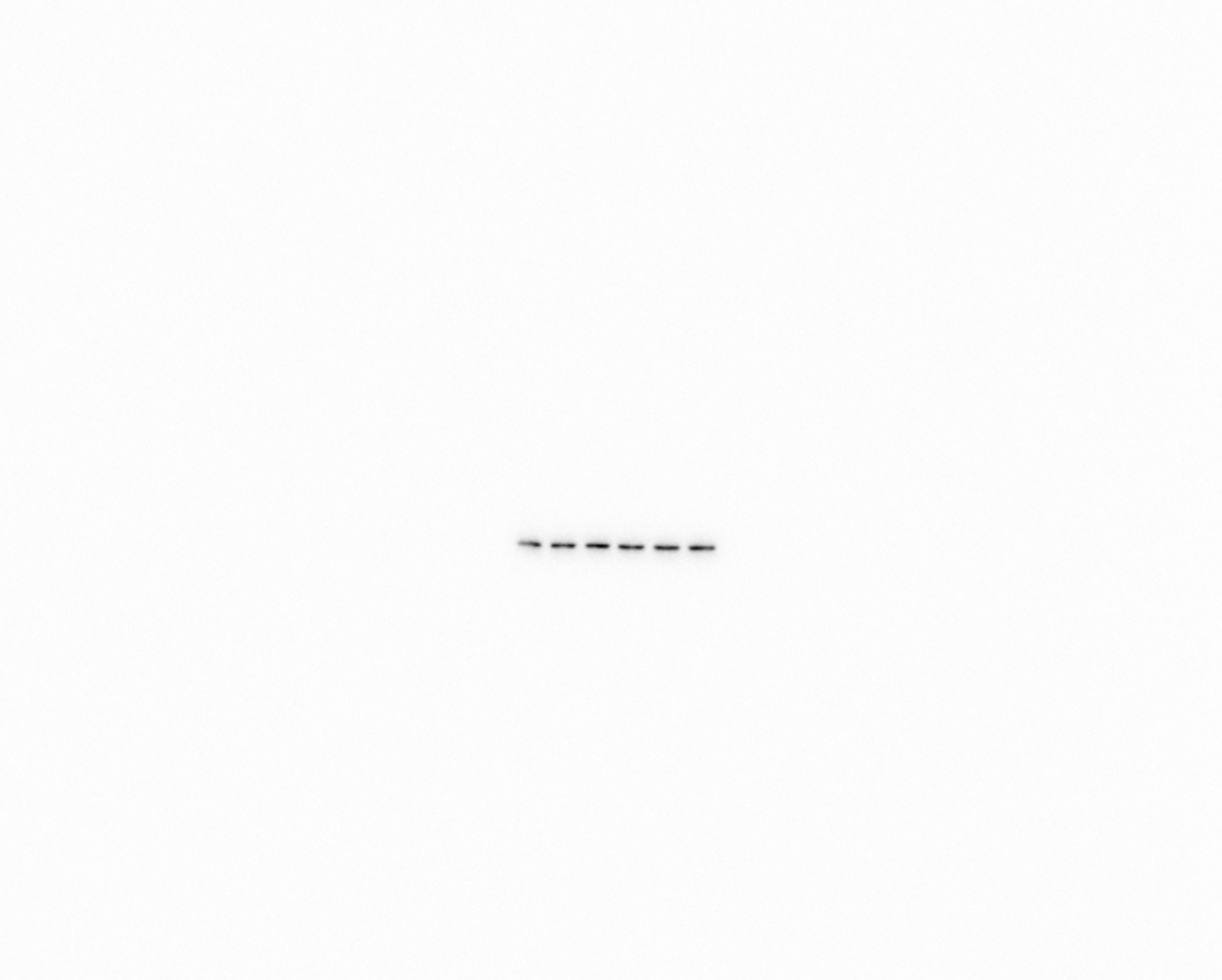


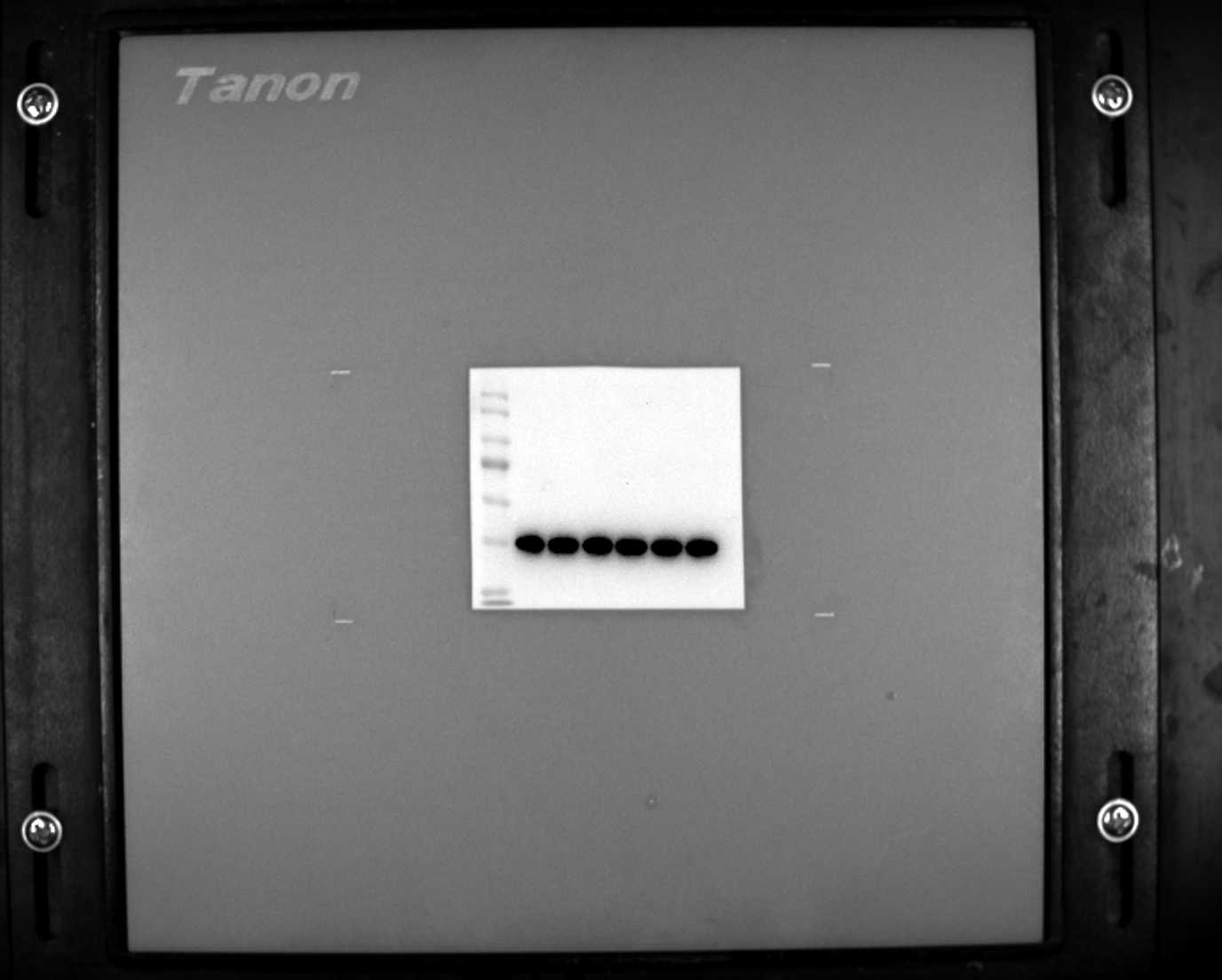

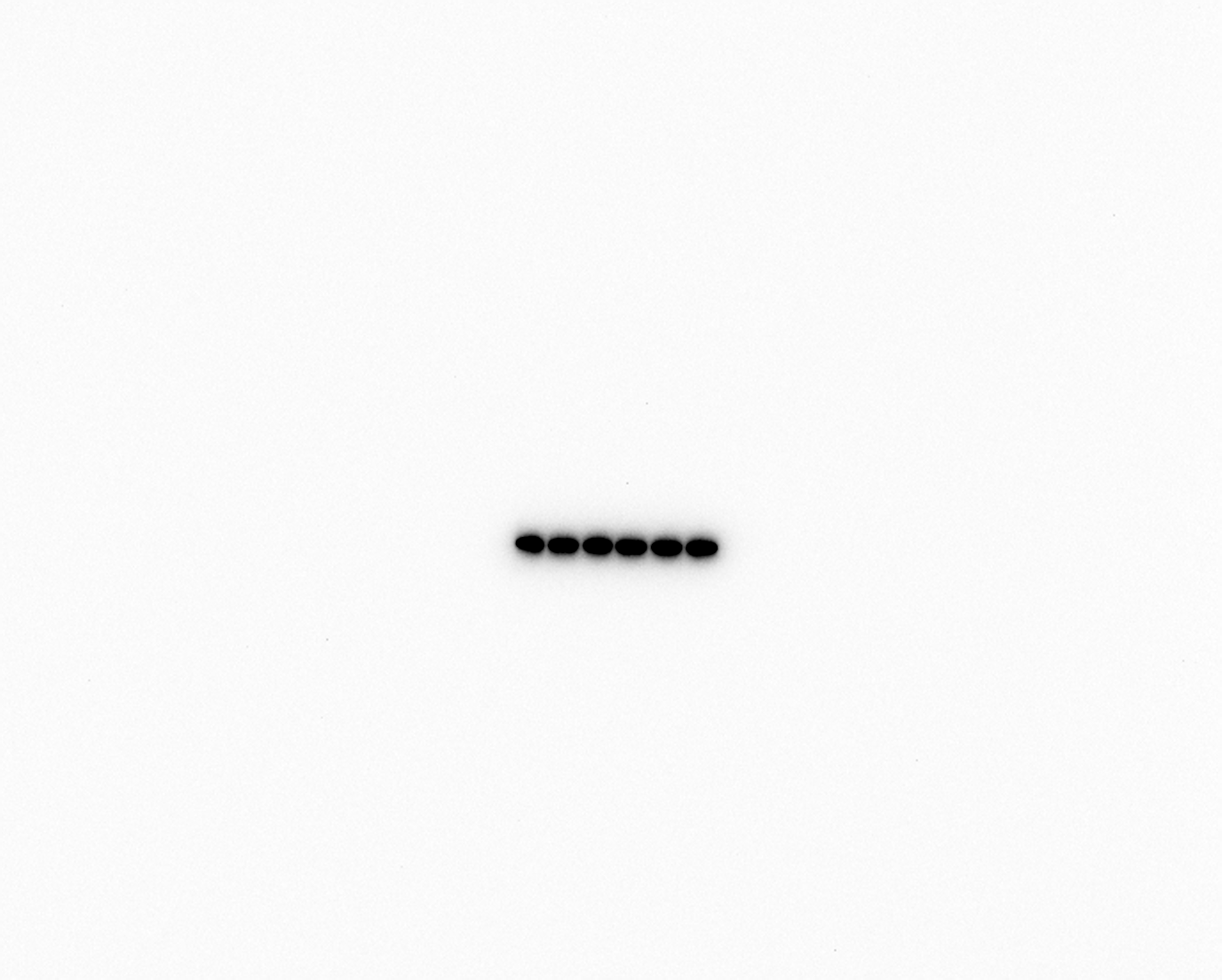


1. Arg1


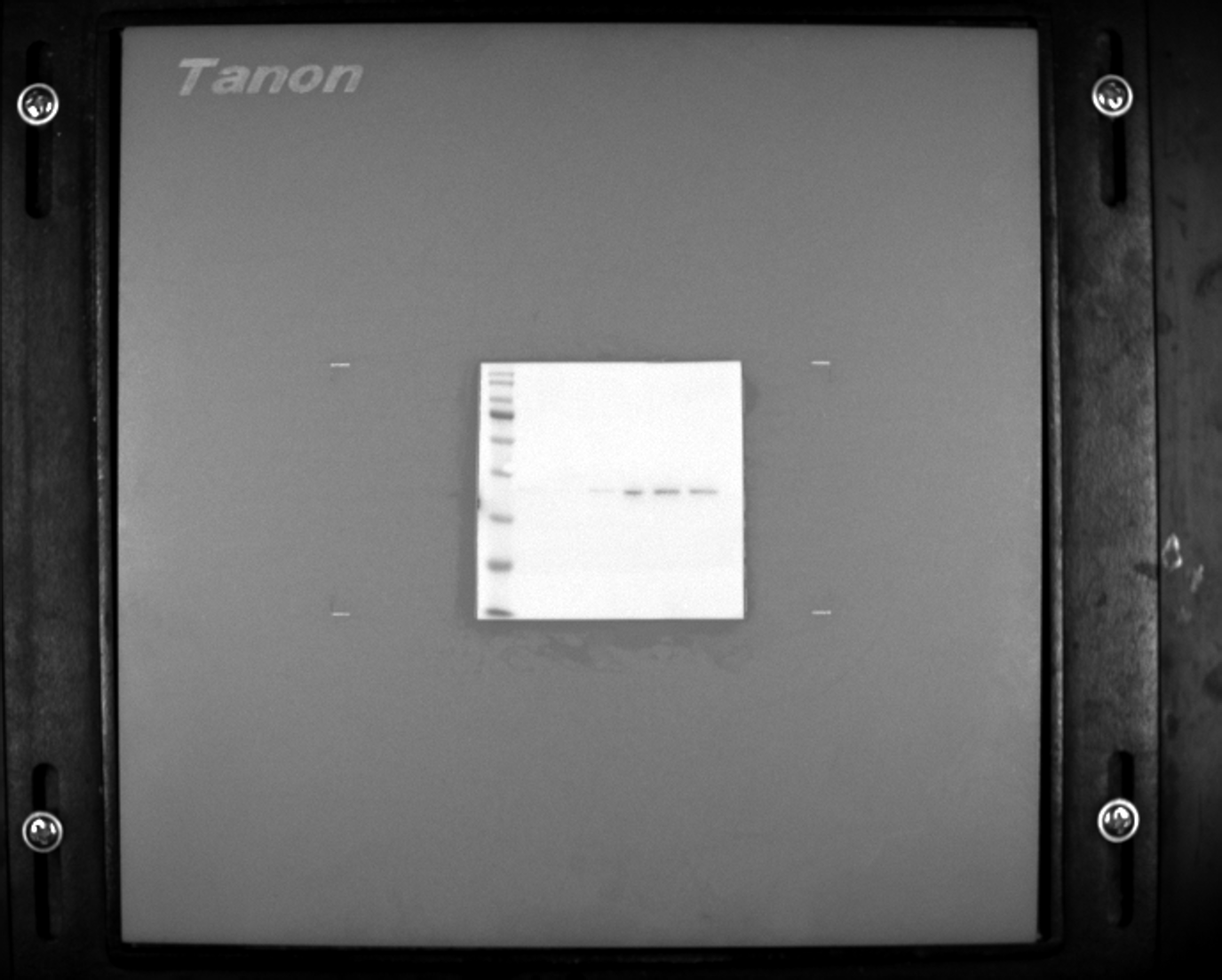

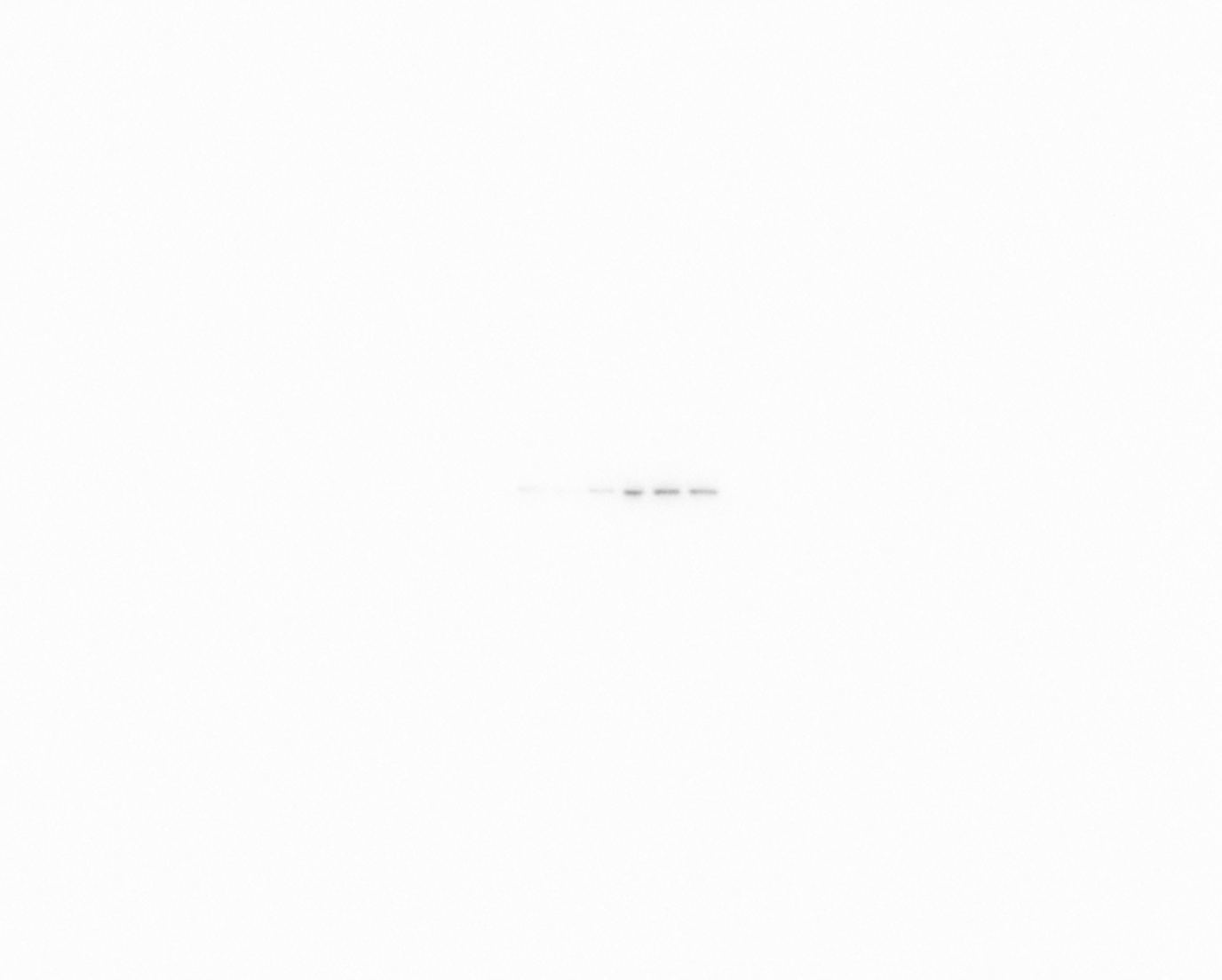


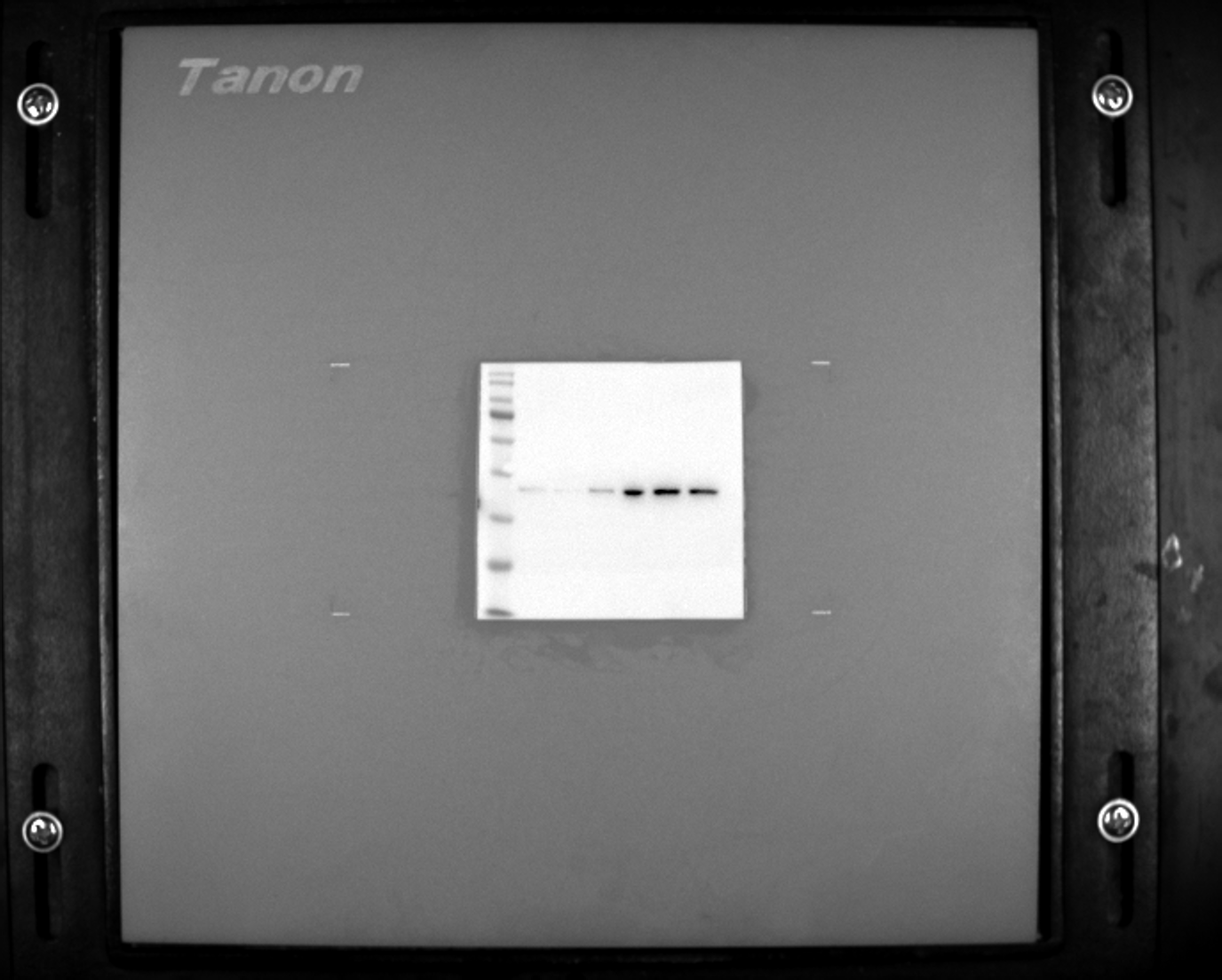

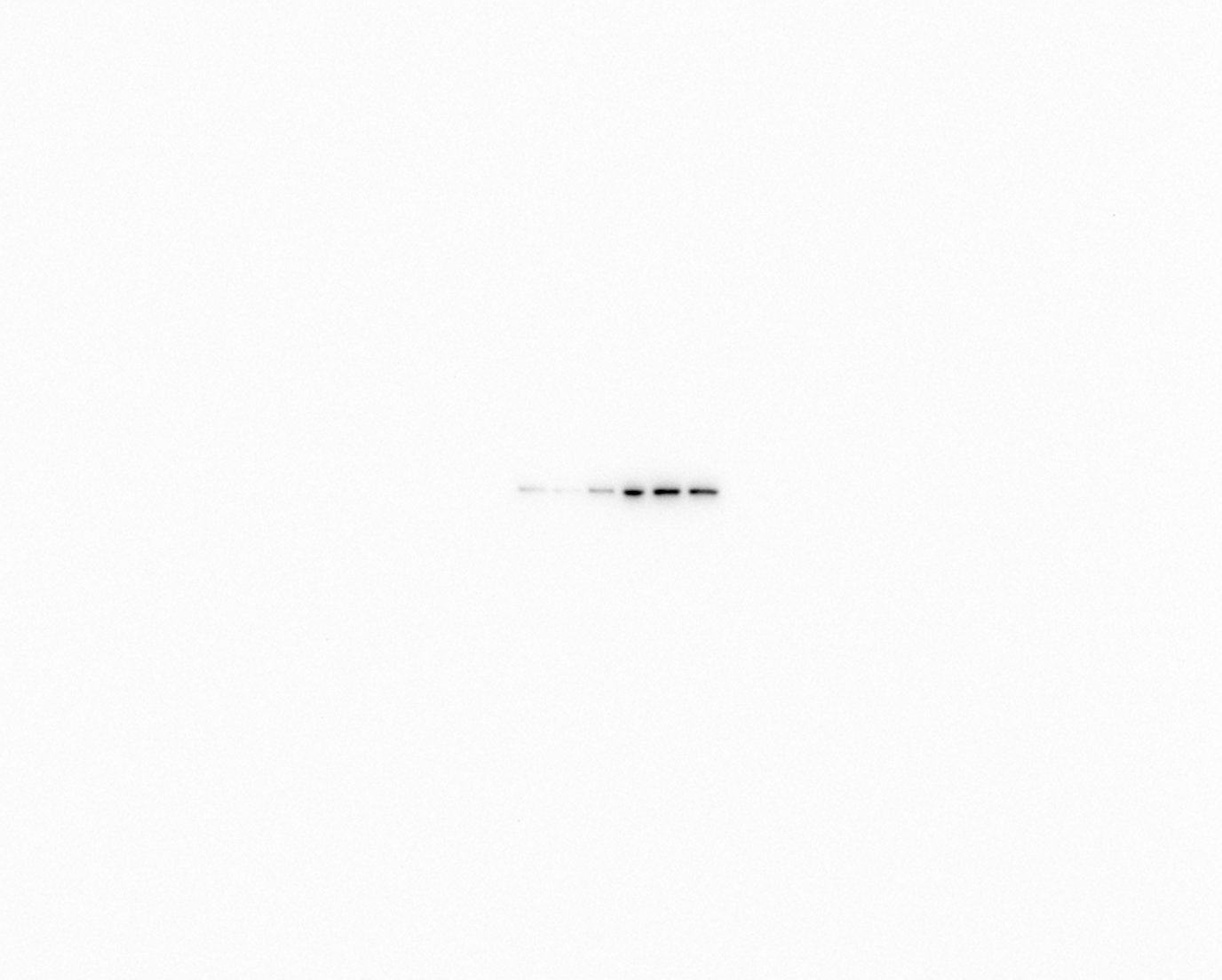

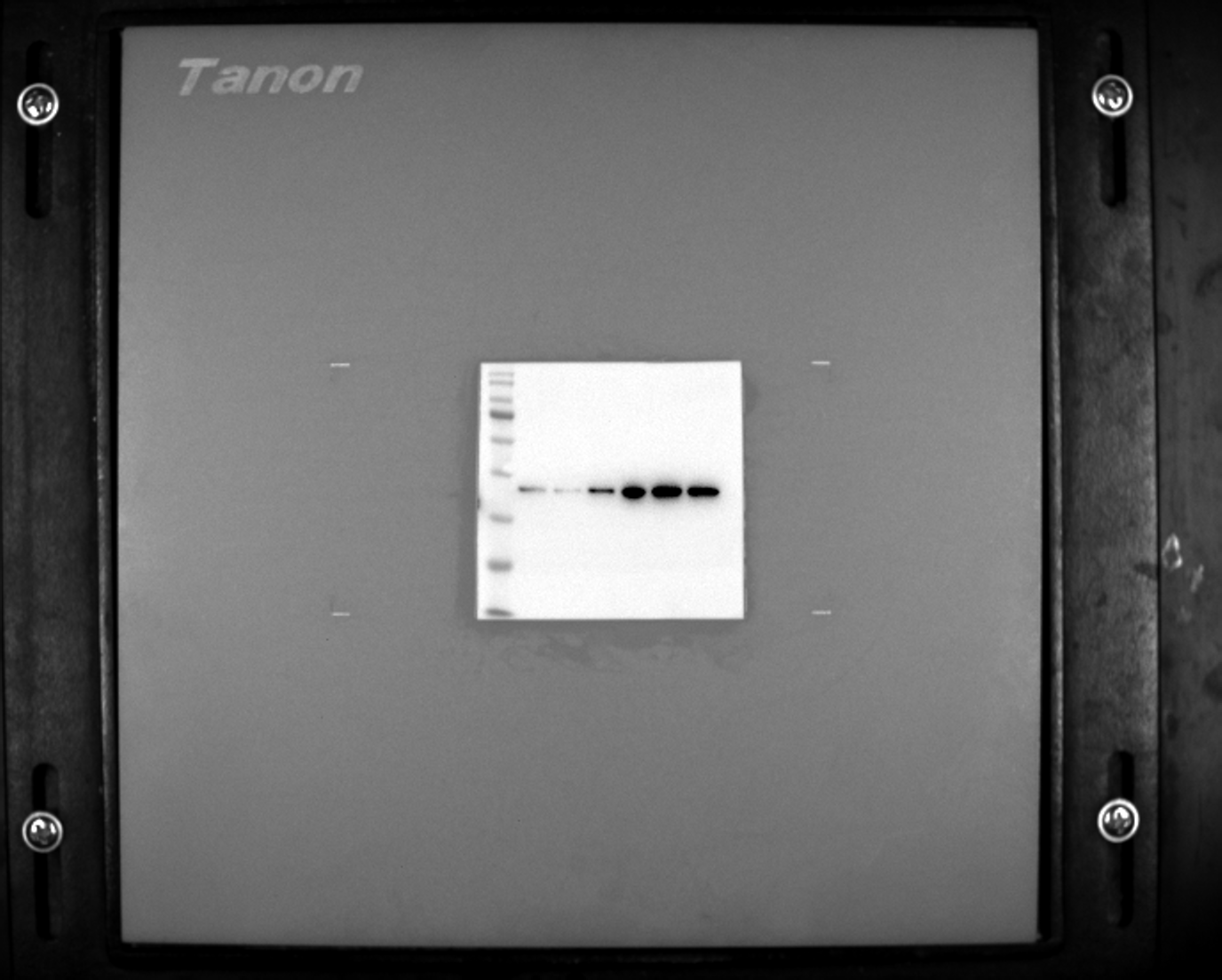

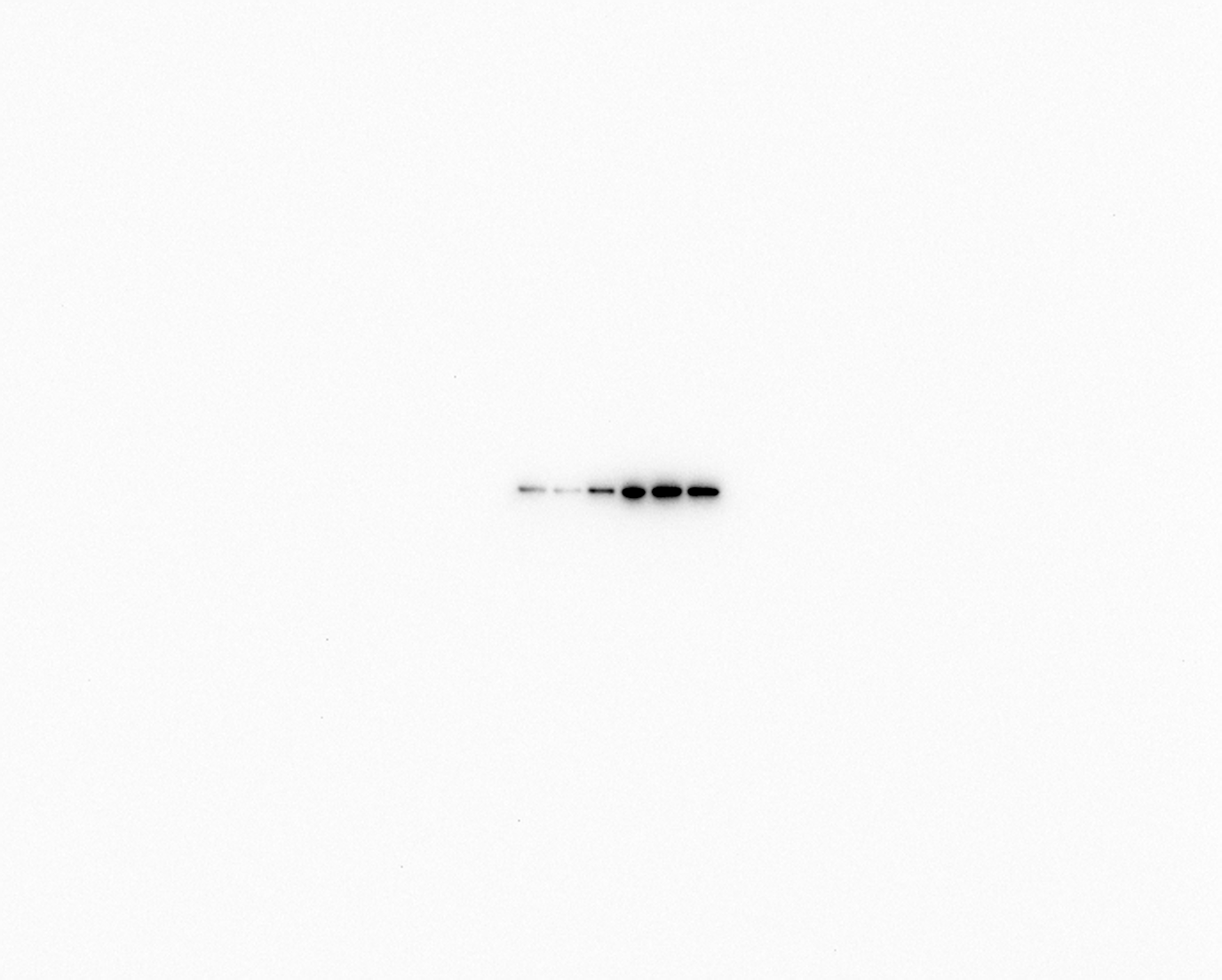


1. CD206


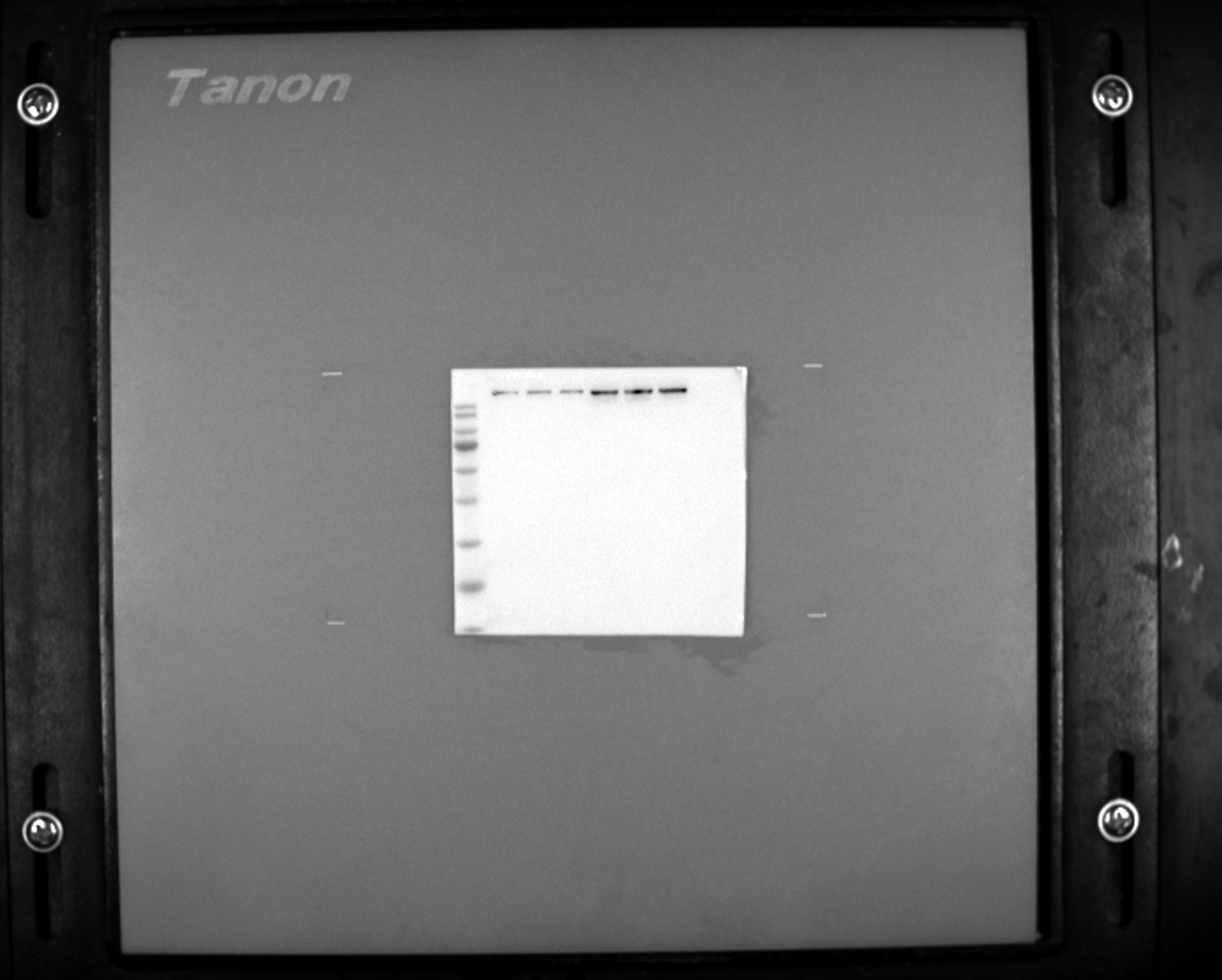

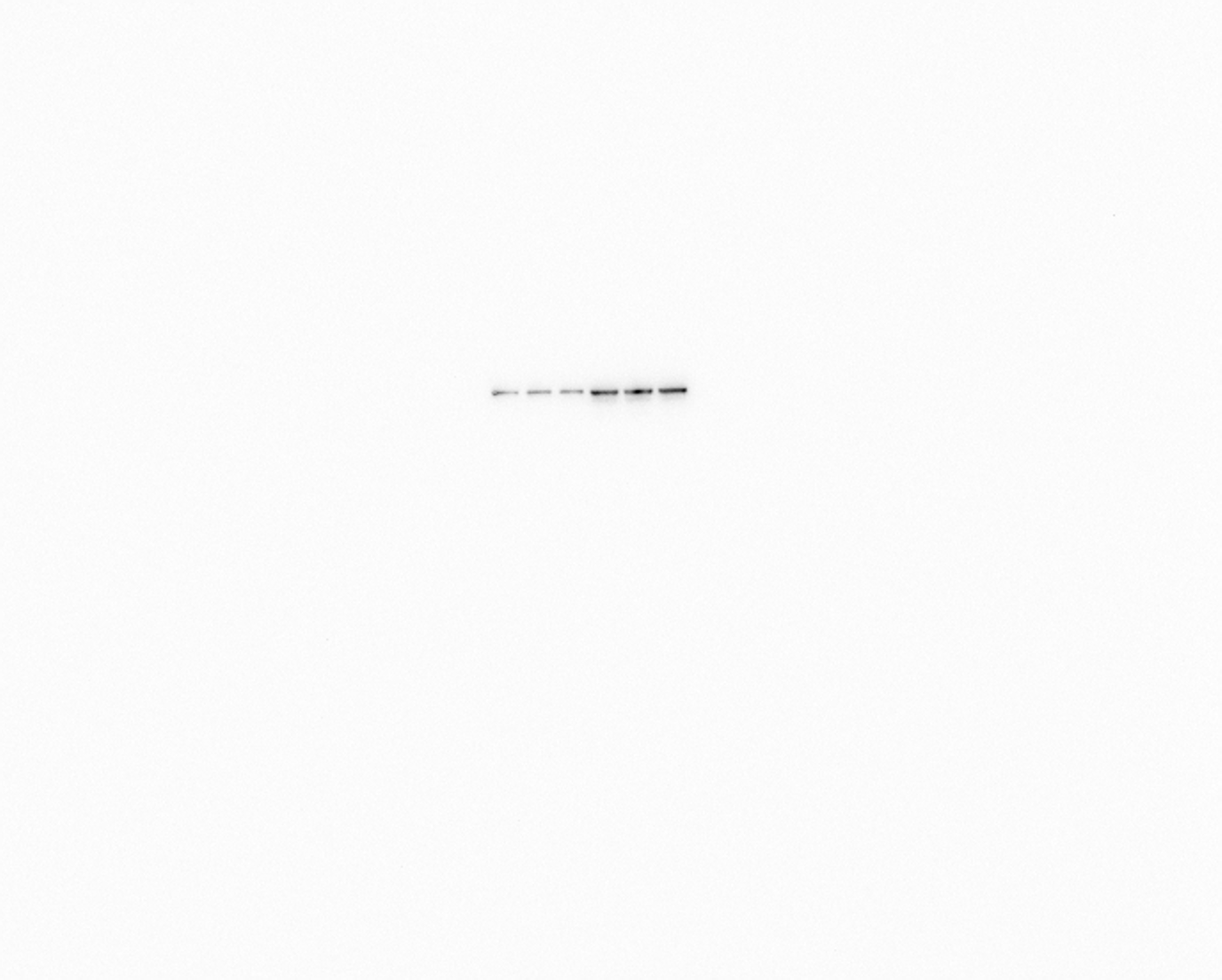

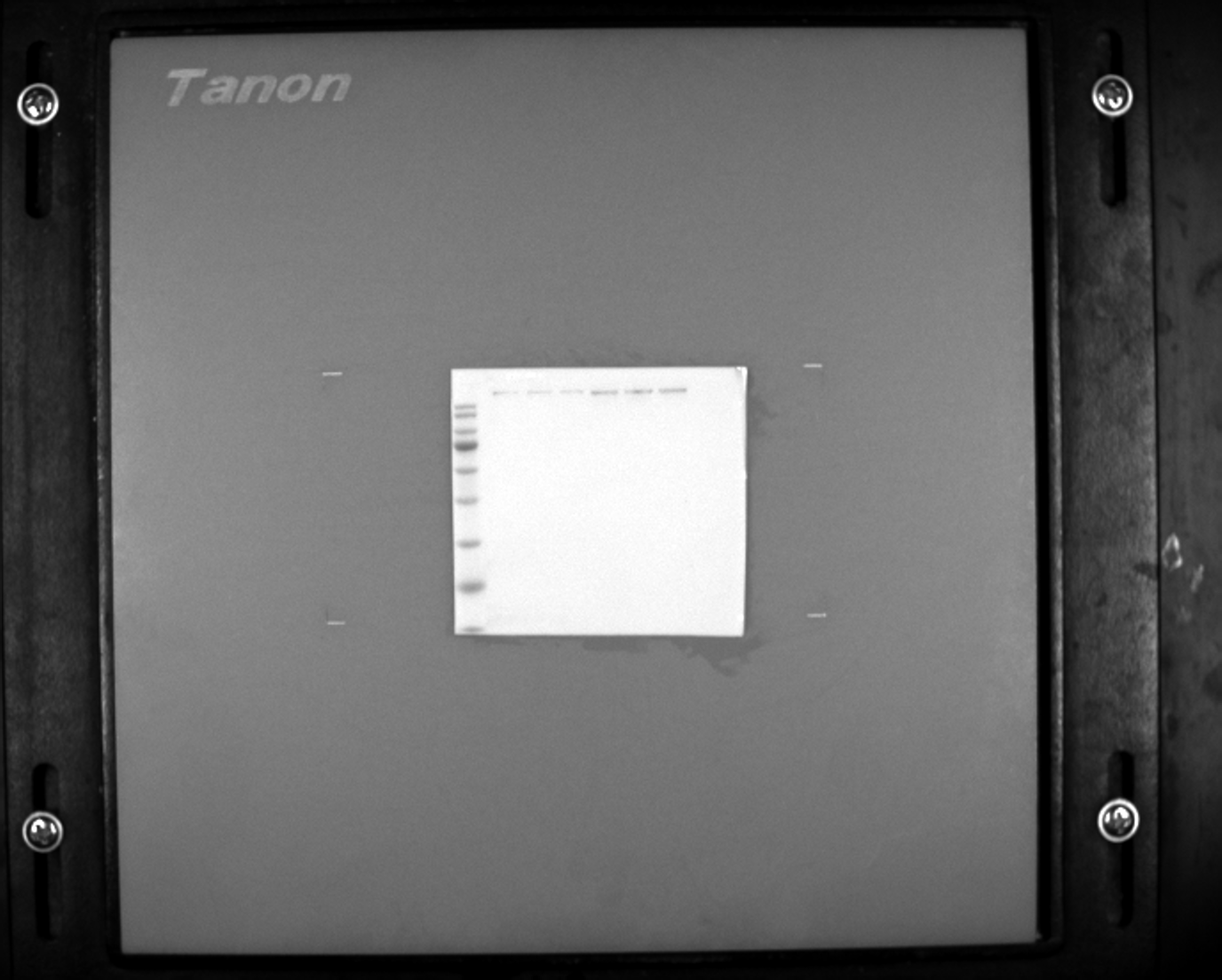

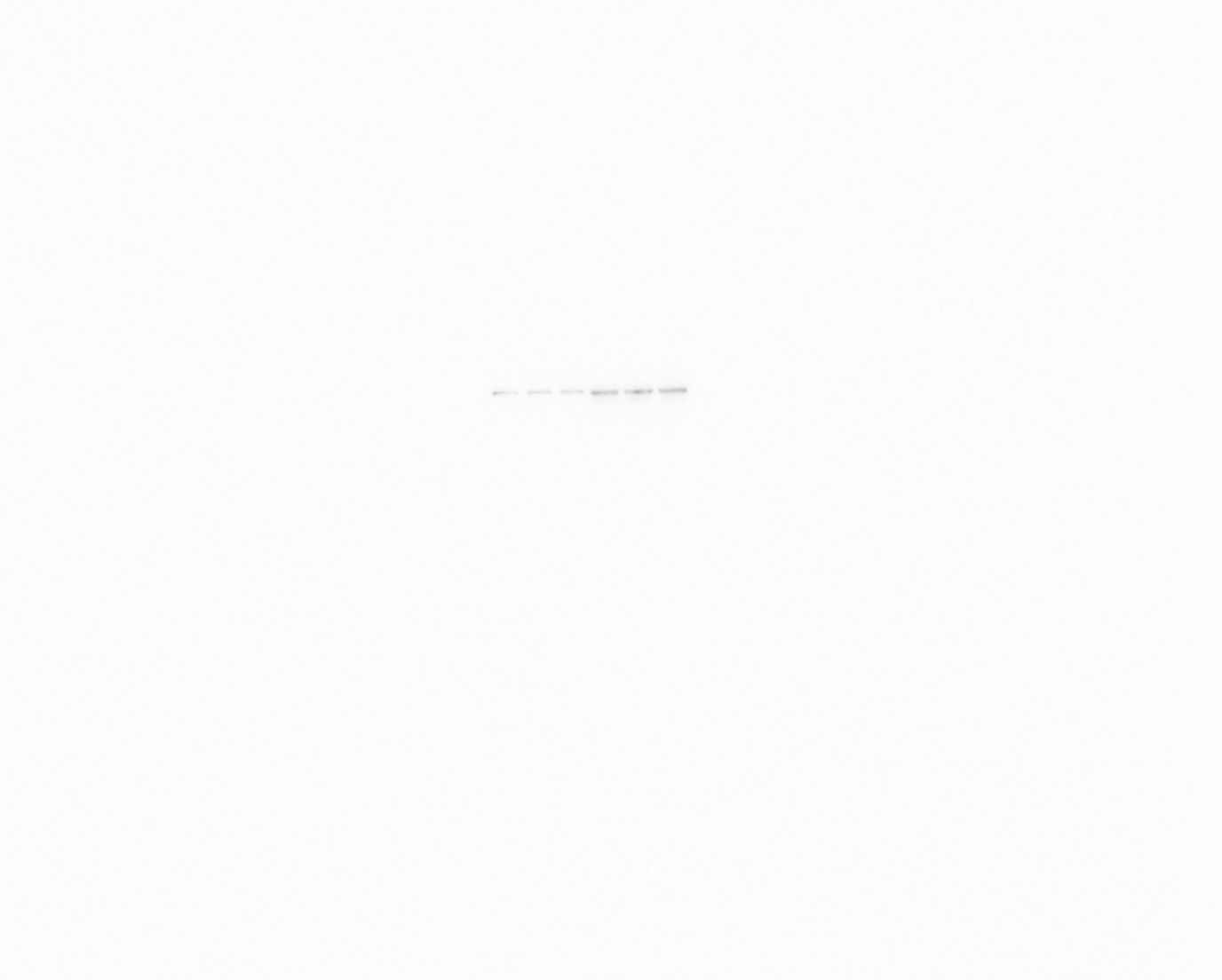

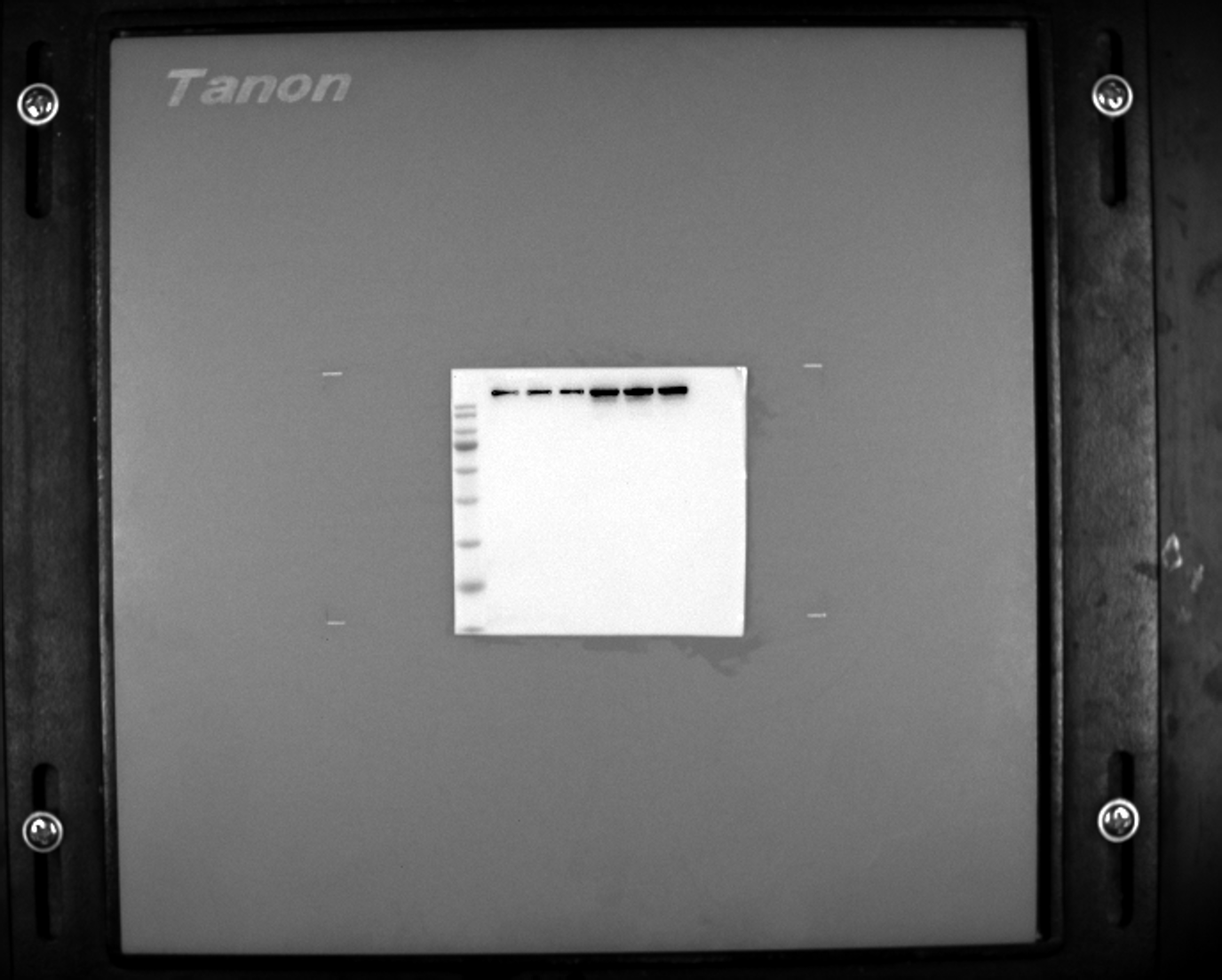

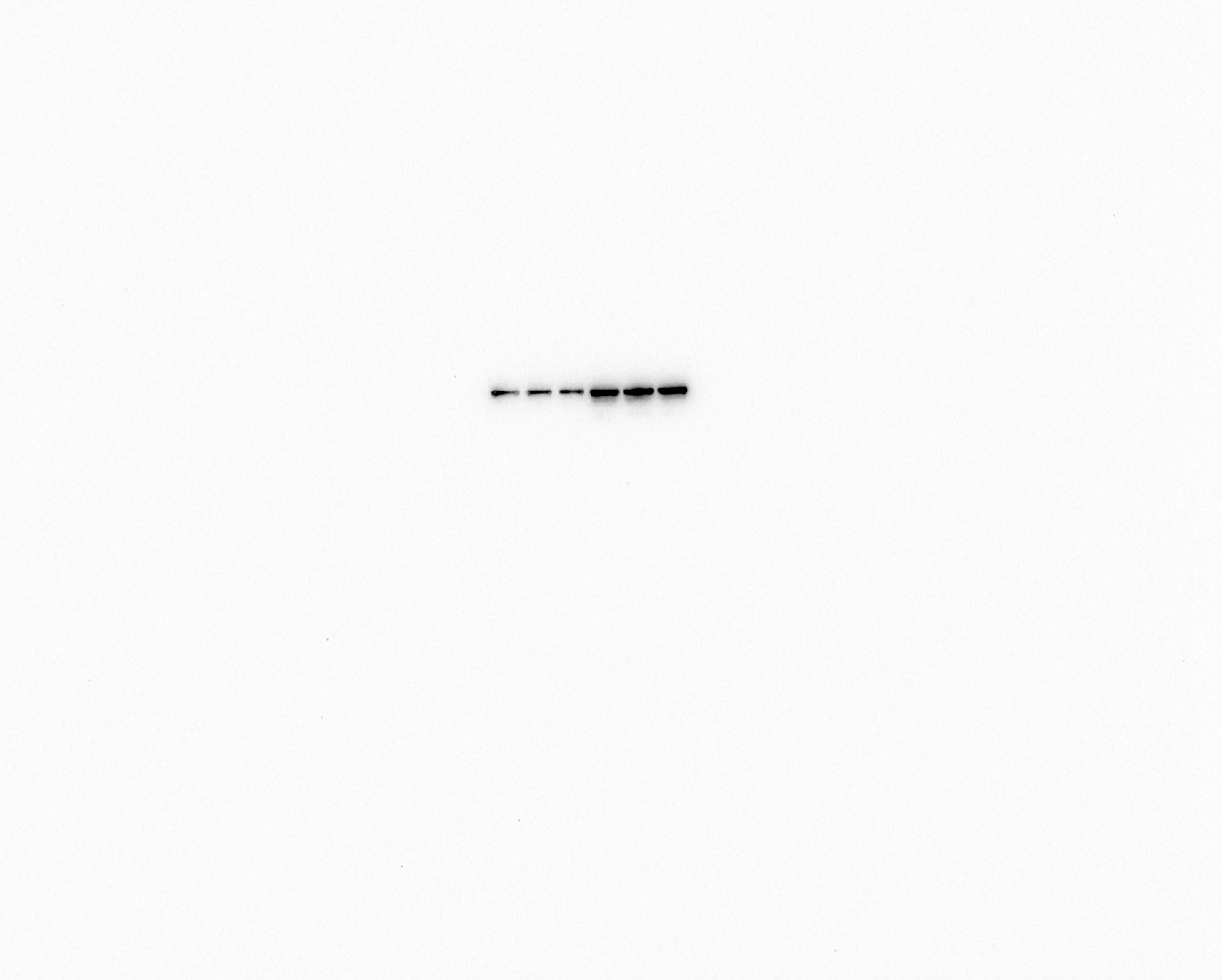


1. iNOS


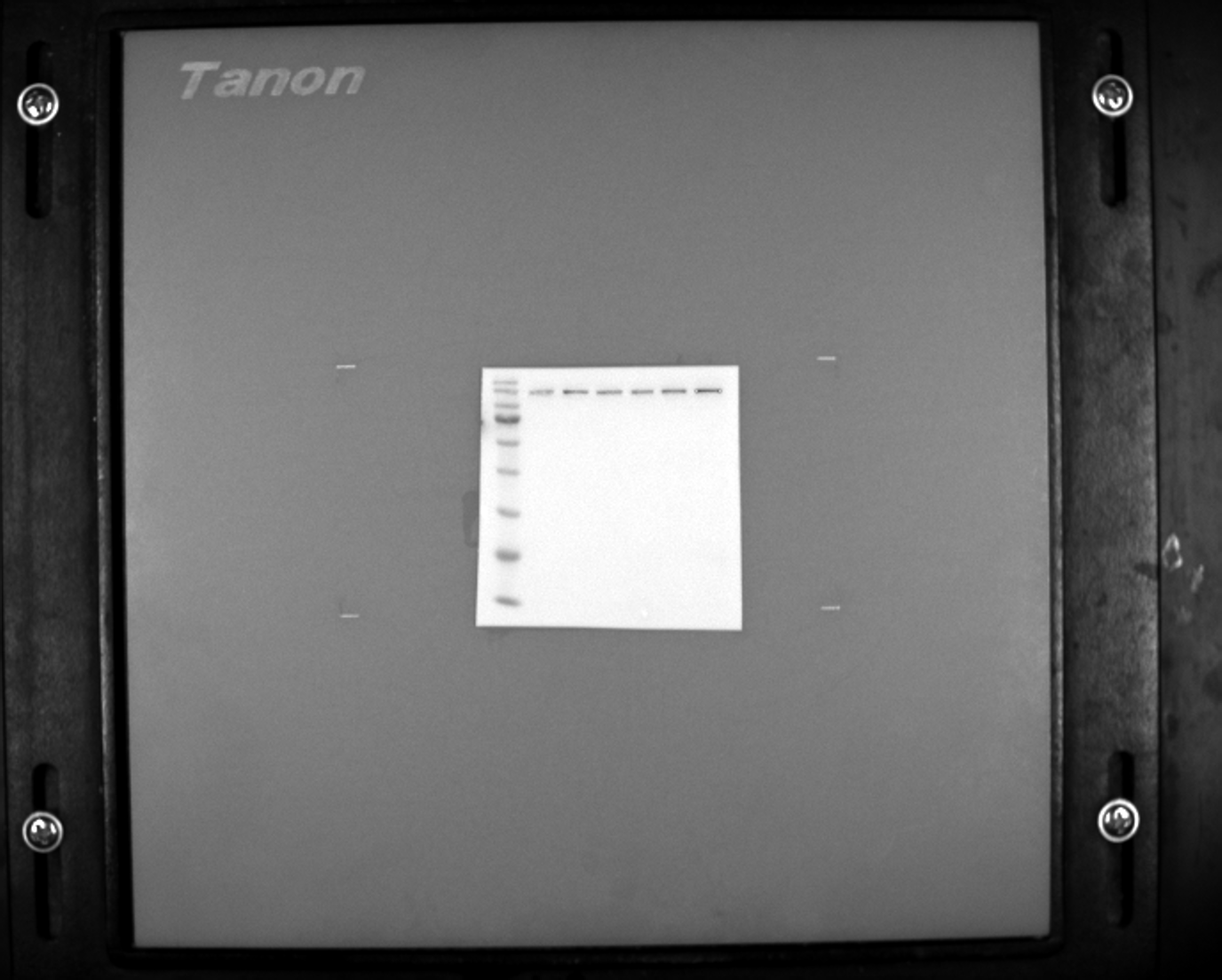

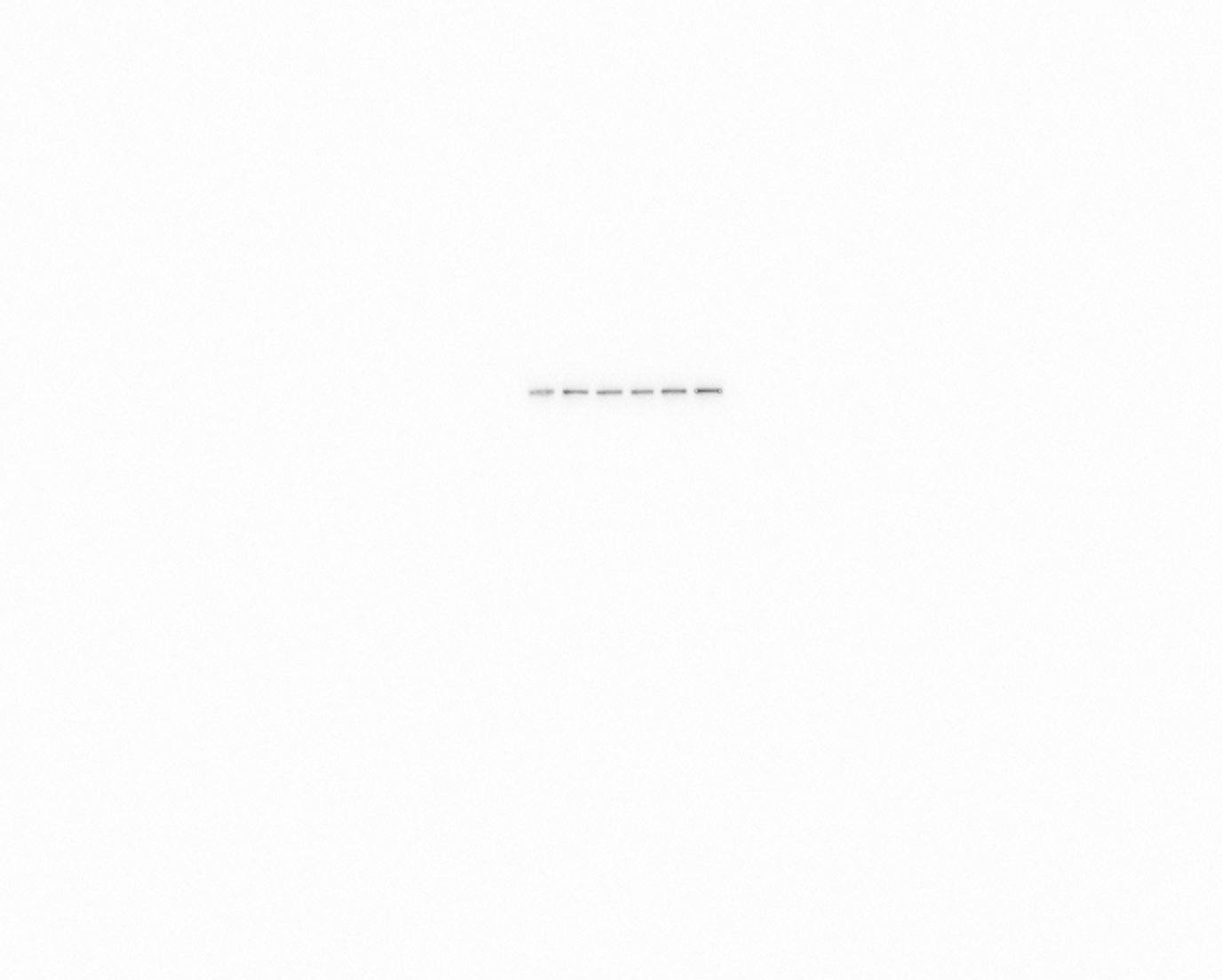

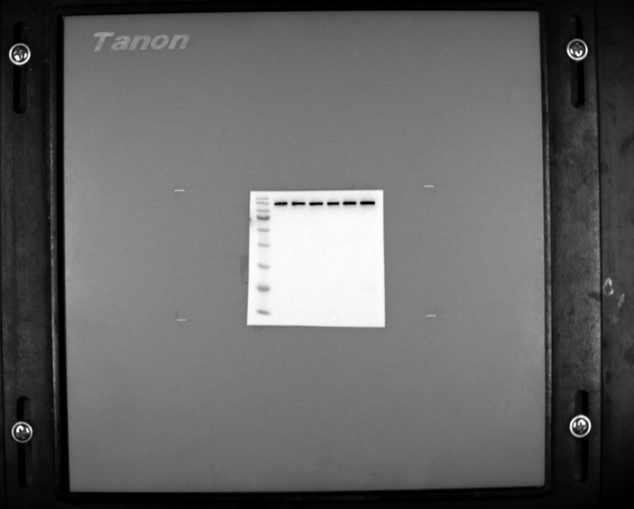

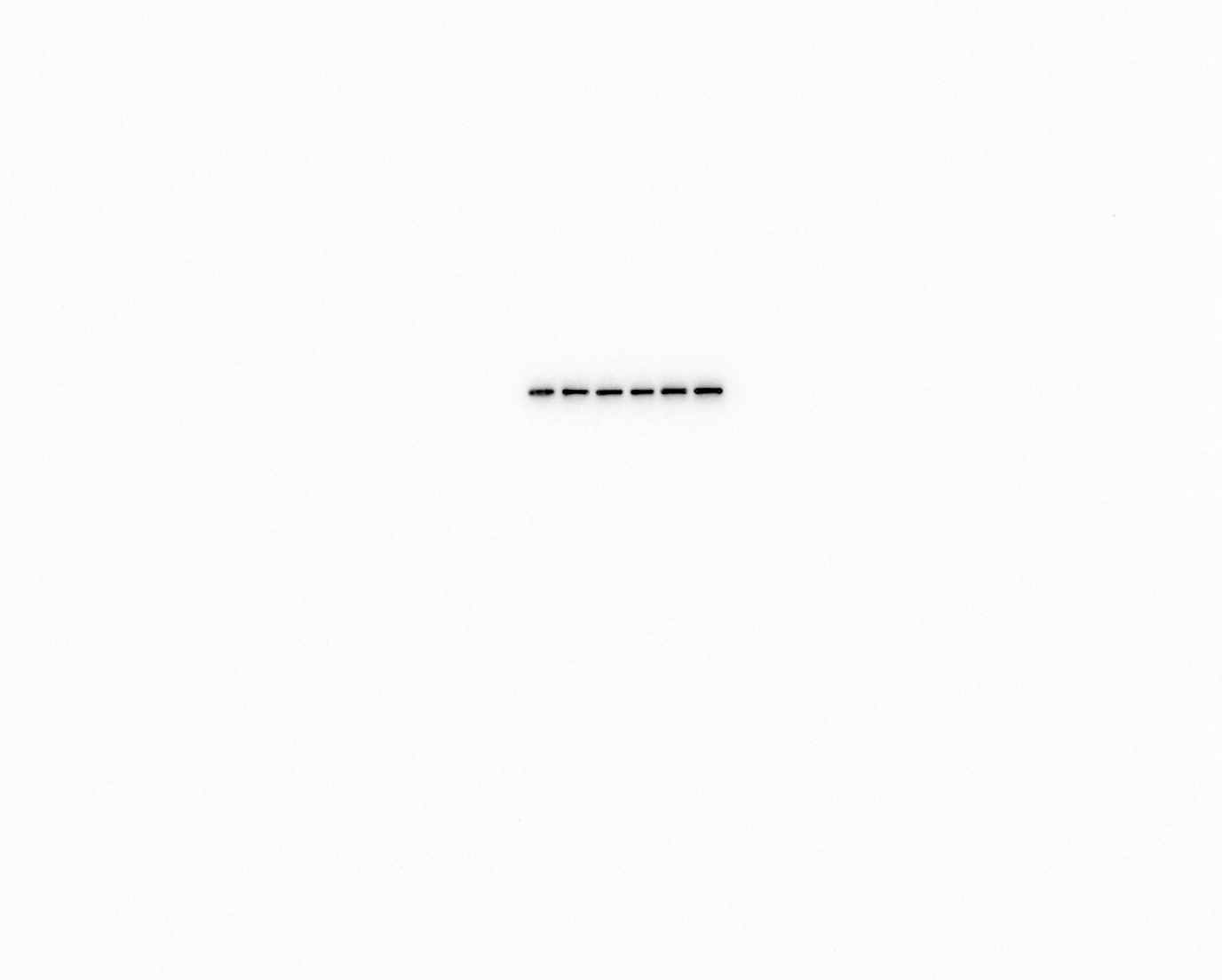

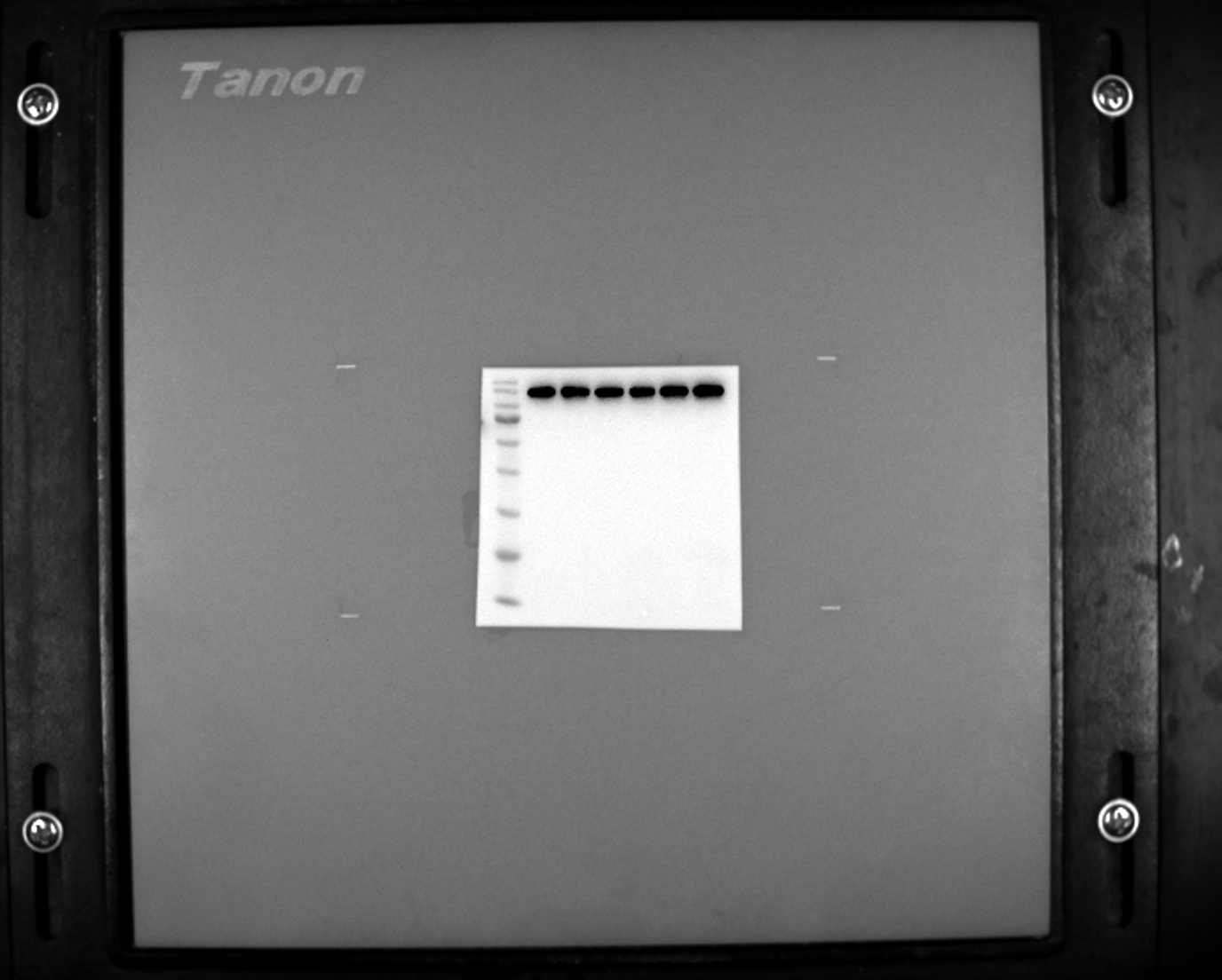

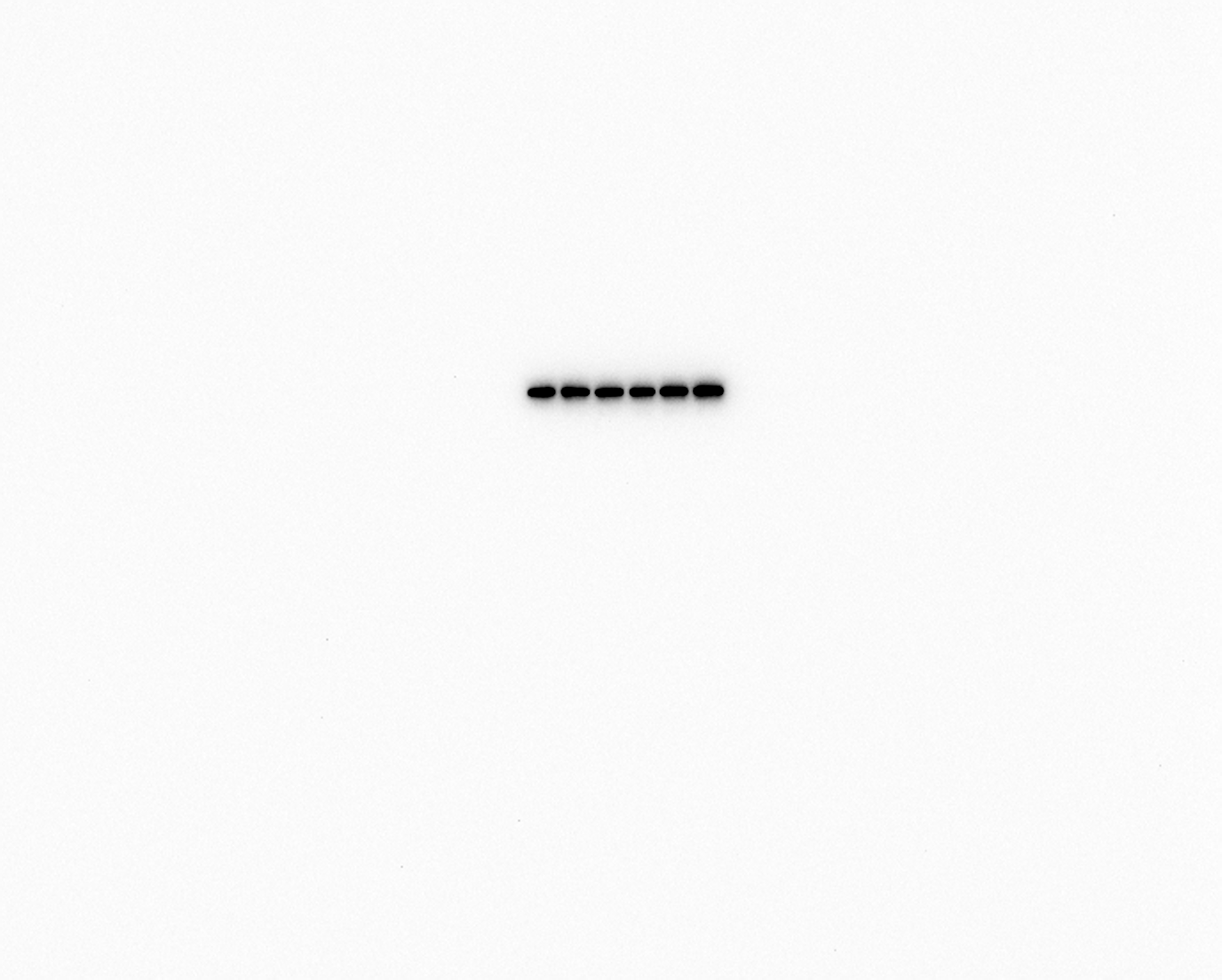


1. IRF5


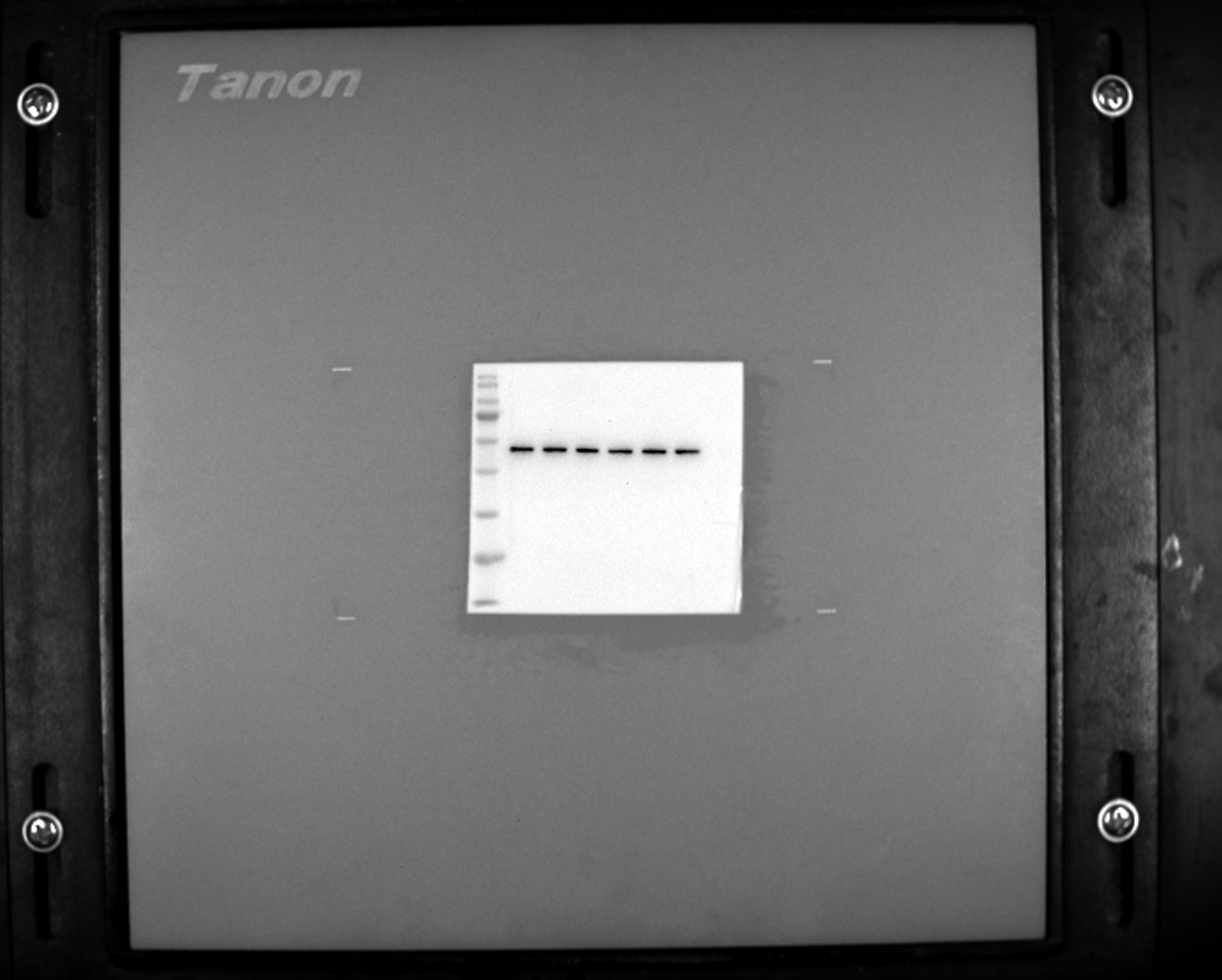

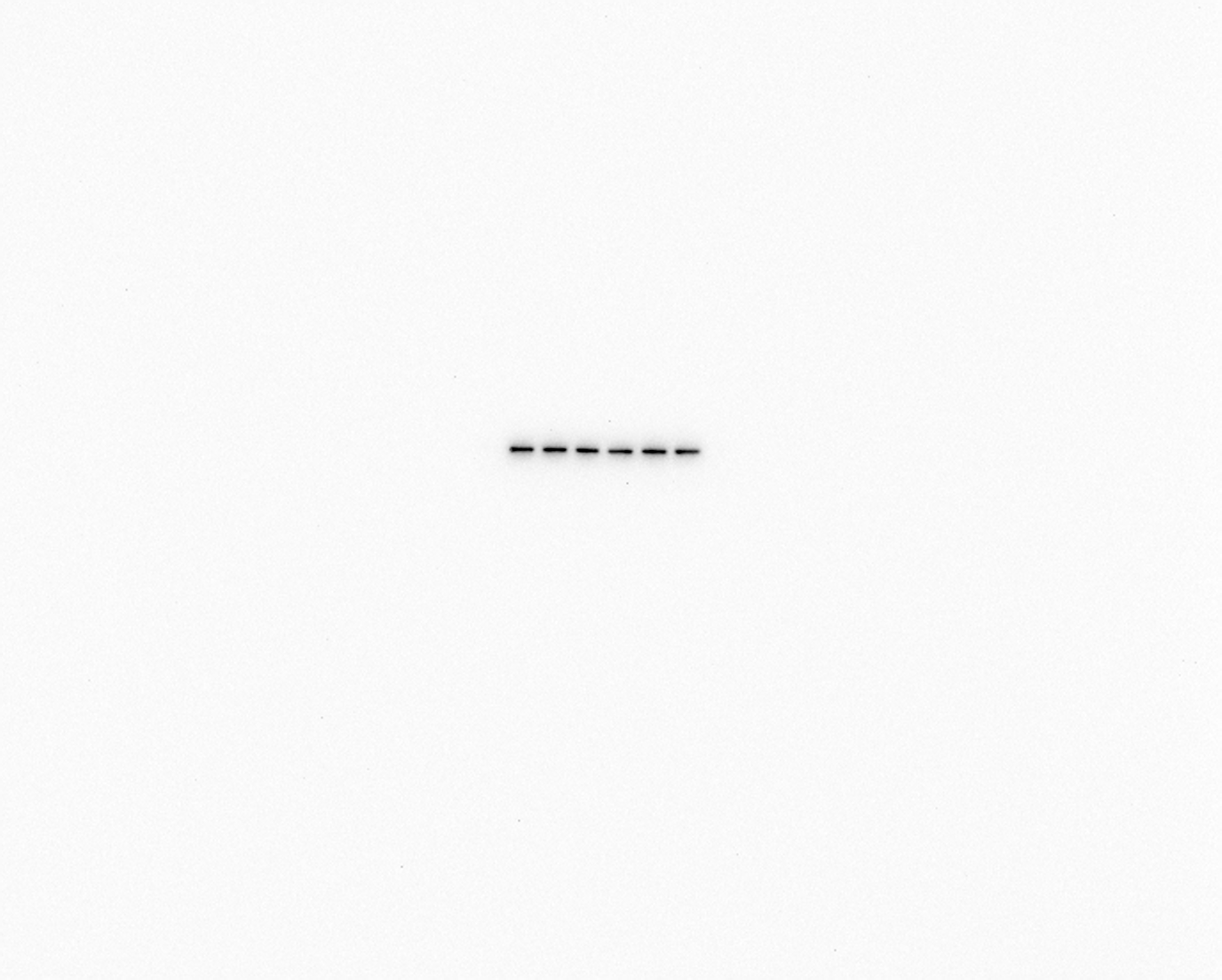

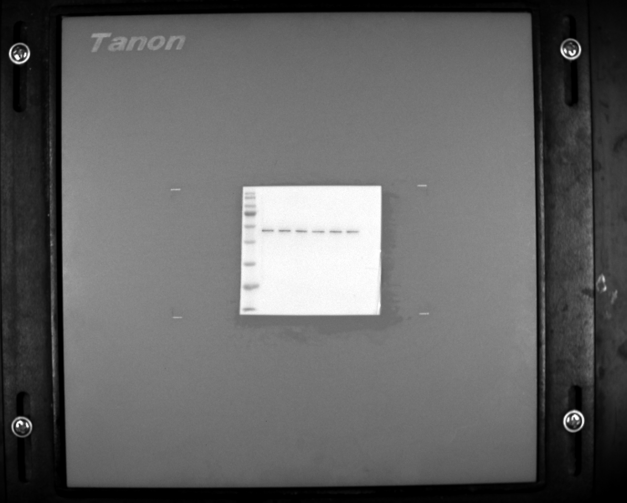

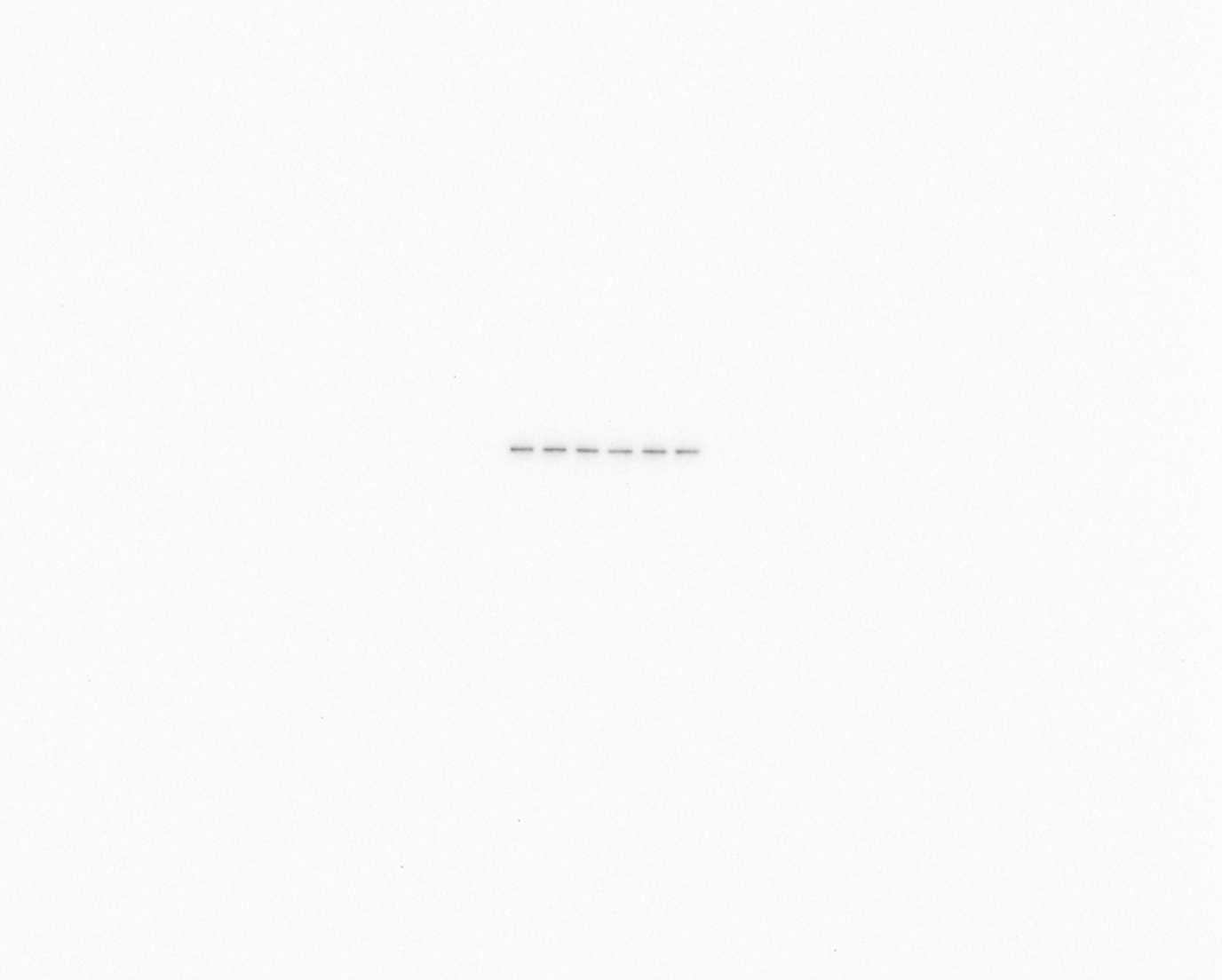

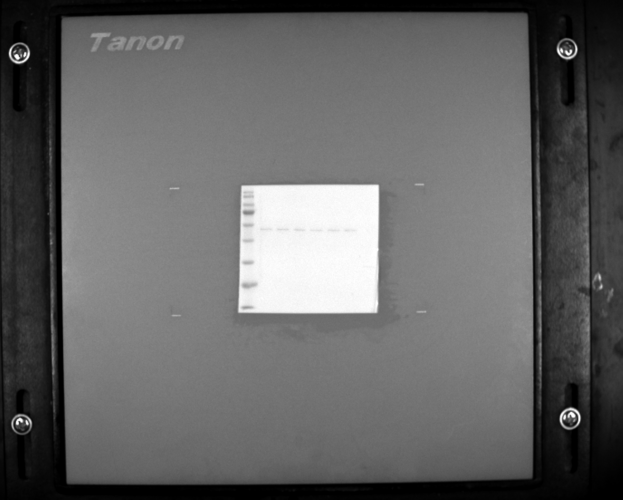

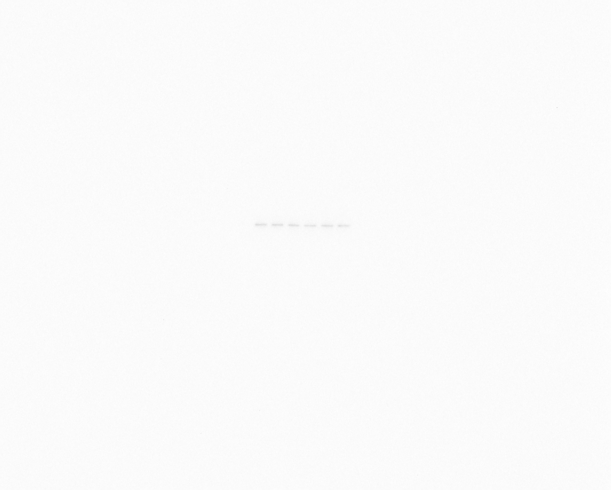

Supplement: Supplementary file 2 — Additional file2 [file 40164_2025_724_MOESM2_ESM.docx]
